# Chronic pharmacologic manipulation of dopamine transmission ameliorates metabolic disturbance in Trappc9-linked brain developmental syndrome

**DOI:** 10.1172/jci.insight.181339

**Published:** 2024-06-18

**Authors:** Yan Li, Muhammad Usman, Ellen Sapp, Yuting Ke, Zejian Wang, Adel Boudi, Marian DiFiglia, Xueyi Li

**Affiliations:** 1 School of Pharmacy, Shanghai Jiao Tong University, Shanghai, China.; 2 Department of Neurology, Massachusetts General Hospital and Harvard Medical School, Charlestown, Massachusetts, USA.

**Keywords:** Metabolism, Neuroscience, Intellectual disability, Mouse models, Obesity

## Abstract

Loss-of-function mutations of the gene encoding the trafficking protein particle complex subunit 9 (Trappc9) cause autosomal recessive intellectual disability and obesity by unknown mechanisms. Genome-wide analysis links Trappc9 to nonalcoholic fatty liver disease (NAFLD). Trappc9-deficient mice have been shown to appear overweight shortly after weaning. Here, we analyzed serum biochemistry and histology of adipose and liver tissues to determine the incidence of obesity and NAFLD in Trappc9-deficient mice and combined transcriptomic and proteomic analyses, pharmacological studies, and biochemical and histological examinations of postmortem mouse brains to unveil mechanisms involved. We found that Trappc9-deficient mice presented with systemic glucose homeostatic disturbance, obesity, and NAFLD, which were relieved upon chronic treatment combining dopamine receptor D2 (DRD2) agonist quinpirole and DRD1 antagonist SCH23390. Blood glucose homeostasis in Trappc9-deficient mice was restored upon administering quinpirole alone. RNA-sequencing analysis of DRD2-containing neurons and proteomic study of brain synaptosomes revealed signs of impaired neurotransmitter secretion in Trappc9-deficient mice. Biochemical and histological studies of mouse brains showed that Trappc9-deficient mice synthesized dopamine normally, but their dopamine-secreting neurons had a lower abundance of structures for releasing dopamine in the striatum. Our study suggests that Trappc9 loss of function causes obesity and NAFLD by constraining dopamine synapse formation.

## Abstract 2

Constraint of dopamine synapse formation underlies obesity and nonalcoholic fatty liver disease associated with the brain developmental syndrome caused by mutations of trappc9.

## Graphical abstract

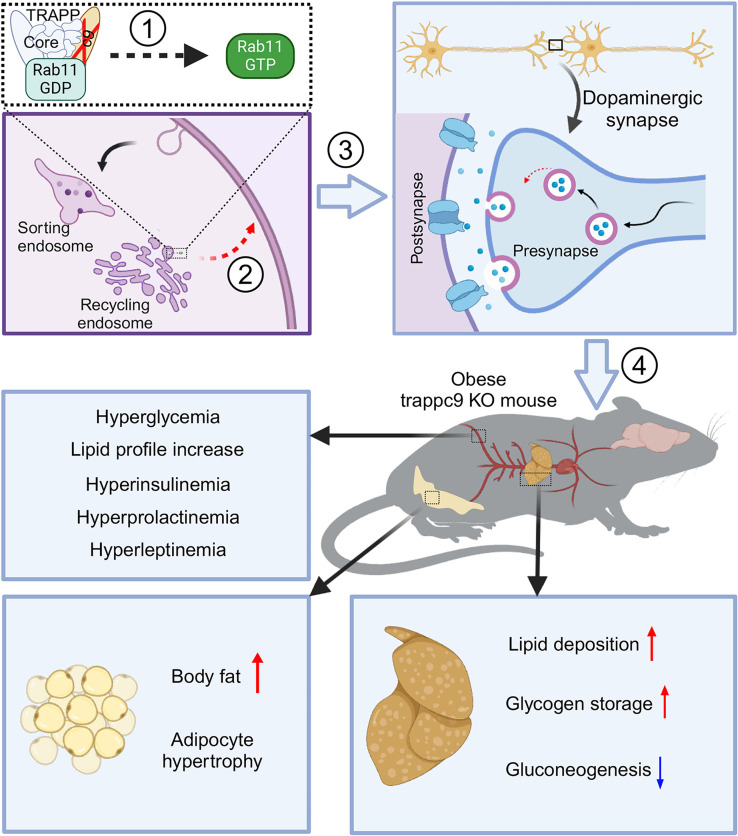

## Introduction

Loss-of-function mutations of the gene coding for the trafficking protein particle (TRAPP) complex subunit 9 (Trappc9) cause an autosomal recessive syndrome with intellectual disability and malformations of various brain structures; over half of the patients exhibit obesity (1–11). Genome-wide analyses uncover differential methylation of the Trappc9 gene in children with severe obesity as well as in women with a high body mass index (12–14) and link Trappc9 to nonalcoholic fatty liver disease (NAFLD) (15), the most common liver disease frequently associated with obesity (16–18). Consistent with a role for Trappc9 in metabolism homeostasis, tracer metabolomics studies detect increased levels of dihydroxyacetone phosphate and glycerol-3-phosphate, which are precursors of triglyceride synthesis, and changes in levels of many other metabolites in skin fibroblasts from patients with the Trappc9 syndrome (11). These human-related studies define Trappc9 as a risk factor for obesity. However, it is not clear how Trappc9 genetic variations trigger the development of obesity and NAFLD.

We and others recently generated genetically modified mice with a loss-of-function mutation in the mouse Trappc9 gene; homozygous mutant or knockout (KO) mice of all these mouse lines show deficits in a wide range of behaviors and malformation of various brain structures replicating what happens in patients (19–22). All these Trappc9-KO mouse lines develop overweight postnatally; female KO mice become overweight earlier than male KO mice (19–22). Adipose-derived stromal or stem cells (ASCs) cultured from Trappc9-KO mice exhibit deficits associated with multiple cellular pathways and contain abnormally large lipid droplets after being induced to differentiate into adipogenic cells (23). Abnormal accumulation of lipid droplets is also observed in hippocampal neurons cultured from Trappc9-KO mice (22). Combined, these findings suggest that Trappc9 deficiency alters lipid dynamics in both neuronal and non-neuronal cells (ASCs and skin fibroblasts). Hence, it is necessary to investigate whether Trappc9-KO mice are metabolically obese.

Trappc9-KO mice are born at normal body size, but female Trappc9-KO mice become overweight as early as age 4 weeks, 1 week after being weaned at age 3 weeks, whereas male KO mice develop overweight at age 8 weeks (19). Coincidentally, Trappc9-KO mice at age 1 month express lower levels of dopamine receptor D2 (DRD2) and higher levels of DRD1 in the striatum relative to age- and sex-matched WT mice (19). Dopamine transmission plays an important role in body weight control and systemic glucose homeostasis by regulating physical activity and food intake (24–29), implying that altered dopamine signaling may play a causative role in obesity development. On the other hand, chronic consumption of obesogenic diets alters the levels of dopamine receptors in the striatum (30, 31), suggesting that perturbation of the dopamine system is likely secondary to obesity development. It is not clear whether the incidence of increased body weight and the altered expression of dopamine receptors in Trappc9-KO mice is related.

In this study, we examined whether Trappc9-KO mice were bona fide obese and explored the mechanism likely to be involved. Our studies showed that Trappc9-KO mice presented with systemic glucose homeostatic disturbances, obesity, and NAFLD. Gene expression analysis of ASCs suggested the involvement of dopamine transmission. Chronic manipulation of dopamine transmission counteracted abnormal body weight gain and alleviated lipid accumulation in adipose and liver tissues in Trappc9-KO mice. Transcriptomic analysis of DRD2-containing neurons and proteomic study of brain synaptosomes pointed to impaired neurotransmitter secretion. Biochemical and histological studies of mouse brain tissues revealed that Trappc9-KO mice had a normal capacity in synthesizing dopamine in the brain, but their dopamine-secreting neurons had a reduced abundance of structures for releasing dopamine in the striatum. Our study suggests that Trappc9 gene mutations trigger the development of obesity by reducing dopamine synapse formation and provides a potential treatment.

## Results

### Trappc9 deficiency increases body adiposity and disturbs the homeostasis of systemic glucose in mice.

Obesity is frequently seen in patients with Trappc9 mutations (11). Genome-wide analysis detects differential methylation in the Trappc9 gene of severely obese children and of women with a high body mass index (12–14). These human studies define Trappc9 as a risk factor for obesity. Consistently, all 4 Trappc9 mutant mouse lines available to date appear overweight postnatally (19–22). However, how Trappc9 mutations cause obesity is an enigma. As Trappc9-KO mice are born with a normal body size and become overweight starting as early as age 4 weeks, 1 week after weaning at age 3 weeks (19), and Liang et al. reported that a patient with Trappc9 syndrome had a history of hyperphagia (20), we monitored food and water intake of Trappc9-KO mice for 1 week. Compared with their age- and sex-matched WT littermates, Trappc9-KO mice were not different in daily intake of water and food (Supplemental Figure 1; supplemental material available online with this article; https://doi.org/10.1172/jci.insight.181339DS1), suggesting that other defects are involved.

**Figure 1:**
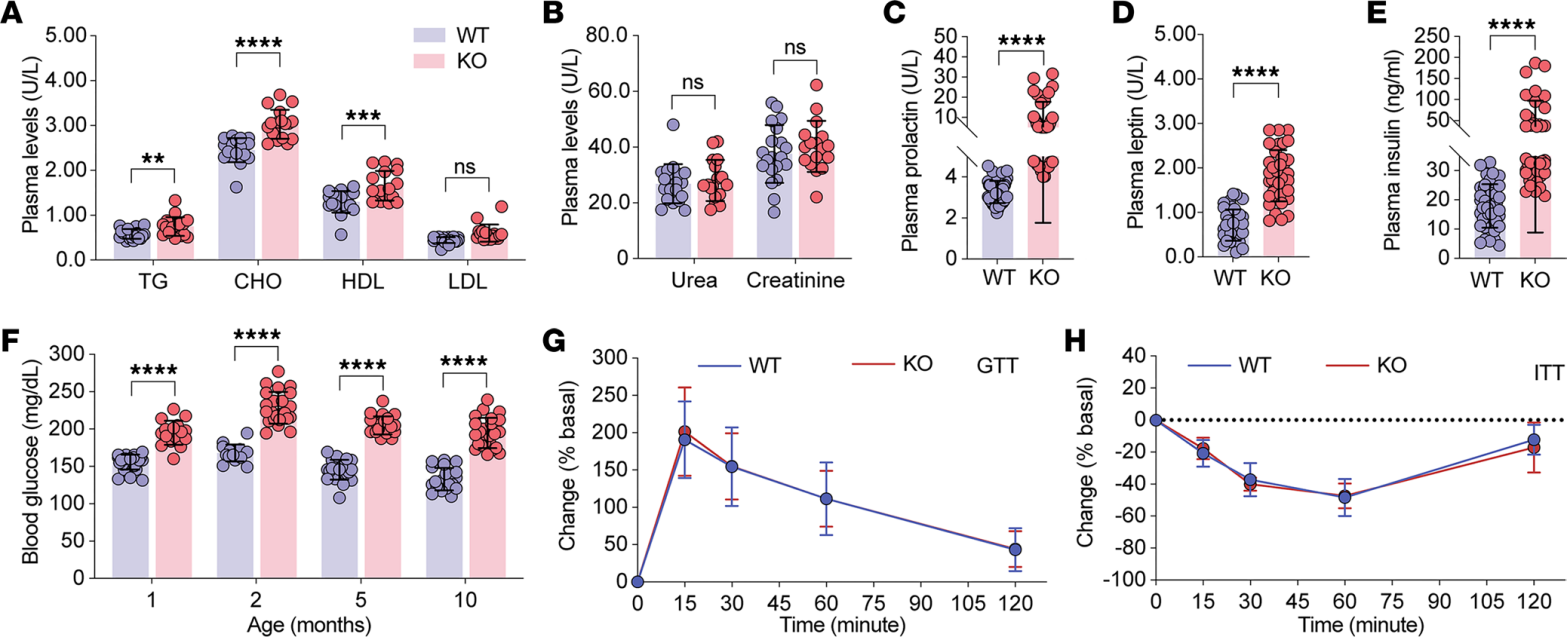
Metabolic disturbance in Trappc9-deficient mice. Serum biochemistry analysis shows that lipid profiles (**A**) were elevated whereas levels of urea and creatinine (**B**) were unchanged in Trappc9-KO mice relative to age-matched WT mice. Trappc9-KO mice also exhibited hyperprolactinemia (**C**), hyperleptinemia (**D**), hyperinsulinemia (**E**), and hyperglycemia (**F**), which were detectable at age 1 month and persisted thereafter. Glucose (**G**) and insulin (**H**) tolerance tests reveal that Trappc9-KO mice responded normally to the challenge with glucose and insulin. Each symbol in bar graphs represents a mouse. *N* = 20 mice (**A**, **B**, and **F**), 30 mice (**C**–**E**), and 12 mice (**G** and **H**) per genotype. Unless indicated, the age of mice was 5 to 6 months. Data are mean ± SD. Two-tailed Student’s *t* test: **P < 0.01; ***P < 0.005; ****P < 0.001. CHO, cholesterol; GTT, glucose tolerance test; ITT, insulin tolerance test.

**Figure 2:**
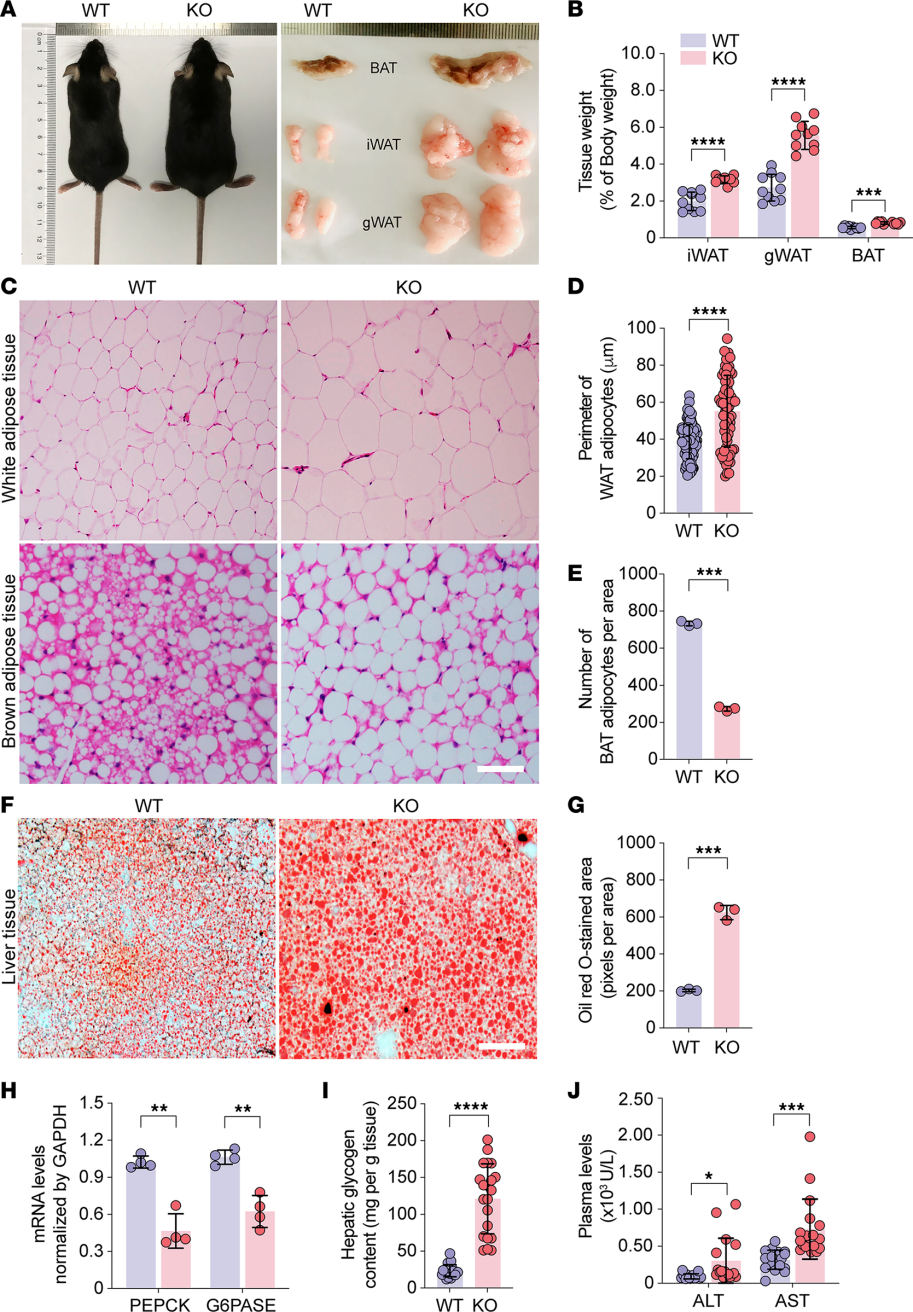
Trappc9-deficient mice develop obesity and NAFLD. (**A**) Comparison of Trappc9 KO and its littermate and fat tissues dissected from the inguinal (iWAT), gonadal (gWAT), and interscapular (BAT) compartments. (**B**) Comparison of fat tissue weight in percentage of body weight between Trappc9-KO and WT mice. (**C**) Hematoxylin and eosin staining of adipose tissues followed by measuring the perimeter (**D**) of adipocytes in white adipose tissues and the number (**E**) of adipocytes in brown adipose tissues. (**F**) Oil Red O staining of liver tissues followed by densitometric quantification of Oil Red O–stained signals (**G**). (**H**) Quantitative PCR analysis of gene transcripts for phosphoenolpyruvate carboxykinase (PEPCK) and glucose-6-phosphatase (G6PASE) in the liver and (**I**) measurements of hepatic glycogen contents. (**J**) Serum biochemistry analysis of the release of alanine aminotransferase (ALT) and aspartate aminotransferase (AST) into the circulating blood in WT and Trappc9-KO mice. Scale bars (**C** and **F**): 50 μm. Each symbol in bar graphs represents 1 mouse (**B**, **E**, and **H**–**J**) or 1 cell (**D**). Shown (**C** and **F**) are representative images from 1 of 3 mice per genotype. At least 3 sections from different compartments (**C**) or lobes (**F**) were analyzed. The age of mice was 5 to 6 months. Data are mean ± SD. Two-tailed Student’s *t* test: *P < 0.05; **P < 0.01; ***P < 0.005; ****P < 0.001.

To determine whether Trappc9-KO mice were metabolically obese, we first analyzed serum biochemistry profiles. Plasma levels of triglycerides, cholesterol, low-density proteins, high-density proteins, glucose, insulin, prolactin, and leptin were all significantly higher in Trappc9-KO mice at age 4–5 months than in age-matched WT mice, whereas plasma levels of urea and creatinine were normal (Figure 1, A–E). Hyperglycemia was detectable in Trappc9-KO mice at age 1 month and persisted thereafter (Figure 1F). Glucose and insulin tolerance tests showed that Trappc9-KO mice were not different from WT mice in responding to challenges with concentrated glucose and/or insulin (Figure 1, G and H), suggesting that insulin sensitivity is still retained in KO mice at age 4–5 months. We then examined the adipose tissues of WT and Trappc9-KO mice. The degree of deposition of white fat in the inguinal (iWAT) and gonadal (gWAT) compartments and brown fat (BAT) in the interscapular compartment was increased in Trappc9-KO mice relative to age- and sex-matched WT mice (Figure 2, A and B). Histological examination revealed a decrease in the cell density and an increase in the size (perimeter) of adipocytes in adipose tissues of Trappc9-KO mice (Figure 2, C–E). The adipocytes in brown fat of WT mice were multilocular, whereas those in Trappc9-KO mice were predominantly unilocular (Figure 2C). These observations suggest that Trappc9-KO mice are bona fide metabolically obese.

### Trappc9-deficient mice develop NAFLD.

Genome-wide association studies link Trappc9 to NAFLD (15), a common comorbidity of obesity (16–18). Liver enlargement is seen in Trappc9-KO mice (19). To examine whether there was abnormal accumulation of lipids in the liver, liver sections of Trappc9-KO mice and their WT counterparts were stained with Oil Red O (Figure 2F). Quantitative analysis of Oil Red O signals showed a greater degree of lipid deposition in hepatocytes of Trappc9-KO mice than in hepatocytes of WT mice (Figure 2G). Hematoxylin and eosin staining revealed that while hepatocyte vacuolation was more apparent in Trappc9-KO mice than in WT mice, no infiltration of inflammatory cells was noted in the liver of Trappc9-KO mice (Supplemental Figure 2). The liver is the primary organ where excess glucose is stored as glycogen and synthesizes glucose through gluconeogenesis when the level of systemic glucose drops. Quantitative PCR analysis was conducted to examine gene transcripts for glucose-6-phosphatase (G6PASE) and phospho-enolpyruvate carboxykinase (PEPCK), both of which play a critical role in gluconeogenesis, and showed that their transcription was downregulated in the liver of Trappc9-KO mice (Figure 2H). On the other hand, the content of hepatic glycogen was elevated in Trappc9-KO mice relative to that in WT mice (Figure 2I). These findings imply reprogramming of glucose metabolism in the liver of Trappc9-KO mice. The elevated plasma levels of alanine aminotransaminase (ALT) and aspartate aminotransferase (AST) suggest liver damage in Trappc9-KO mice (Figure 2J). Collectively, our results indicate that Trappc9-KO mice have NAFLD, which has not yet progressed into steatohepatitis.

### Pharmacologic manipulation of dopamine transmission restores the homeostasis of circulating glucose.

**Figure 3:**
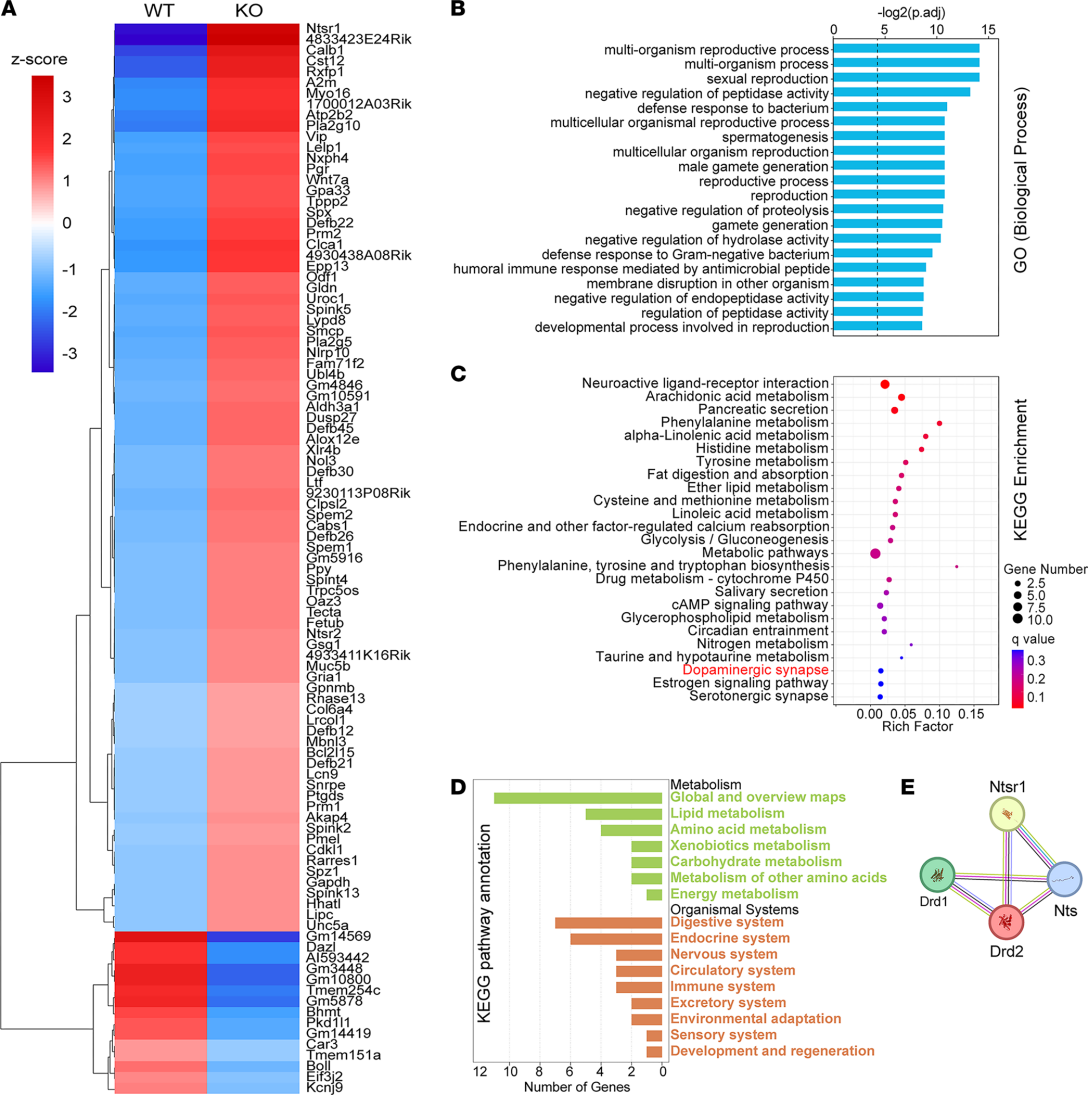
Transcriptomic analysis of ASCs reveals metabolic disturbance and the involvement of dopamine synapse in Trappc9-deficient mice. (**A**) Heatmap shows differentially expressed genes identified by RNA-Seq analysis of ASCs in Trappc9-KO mice. ASCs were isolated from abdominal fat pads of 1-month-old WT and Trappc9-KO mice (*N* = 3 to 5 mice per genotype) and directly subjected to RNA preparations without culture in vitro. GO function (**B**) and KEGG pathway (**C** and **D**) analyses of differentially expressed genes in ASCs of Trappc9-KO mice. Shown (**B** and **C**) are top 25 GO biological processes or KEGG pathways. KEGG pathway annotation shows metabolic disturbances in multiple systems (**D**). (**E**) The STRING functional protein association network shows interplay between the neurotensin and dopamine systems.

Having demonstrated that Trappc9-KO mice develop obesity and NAFLD, we then looked for the underlying mechanisms. ASCs play an important role in the development of obesity by remodeling adipose tissues by generating new adipocytes and secreting bioactive molecules to maintain the immunological homeostasis in adipose tissues (32–35). ASCs cultured from Trappc9-KO mice exhibit defects in multiple cellular pathways and are prone to senescence (23). To identify the molecular changes, ASCs were isolated from abdominal fat pads of 1-month-old Trappc9-KO mice and WT littermates and directly subjected to RNA extraction without culture in vitro. RNA-sequencing (RNA-Seq) analysis identified 15 downregulated and 84 upregulated protein-coding genes in ASCs of Trappc9-KO mice (Figure 3A and Supplemental Table 1). Consistent with our previous finding of infertility of male Trappc9-KO mice, Gene Ontology (GO) function analysis revealed alterations in reproductive processes and spermatogenesis (Figure 3B and Supplemental Table 2). Kyoto Encyclopedia of Genes and Genomes (KEGG) pathway analysis suggested disturbances in carbohydrate, lipid, and amino acid metabolism and the involvement of dopaminergic synapses in Trappc9-KO mice (Figure 3, C and D, and Supplemental Table 3). Neurotensin receptor (NtsR) 1 and 2 were among the top hits of the identified genes differentially expressed in Trappc9-KO ASCs (Figure 3A). STRING functional protein interaction network analysis revealed interplay between the neurotensin and dopamine systems (Figure 3E). The neurotensin system also plays a pivotal role in body weight control and regulates dopamine secretion in the brain (36–39). These data are consistent with our previous finding that dopamine transmission is altered in Trappc9-KO mice (19). As the treatment combining DRD1 antagonist SCH23390 and DRD2 agonist quinpirole is effective in ameliorating deficits associated with multiple behaviors in Trappc9-KO mice (19), we reasoned that this treatment might also be beneficial in controlling obesity or NAFLD in Trappc9-KO mice.

**Figure 4:**
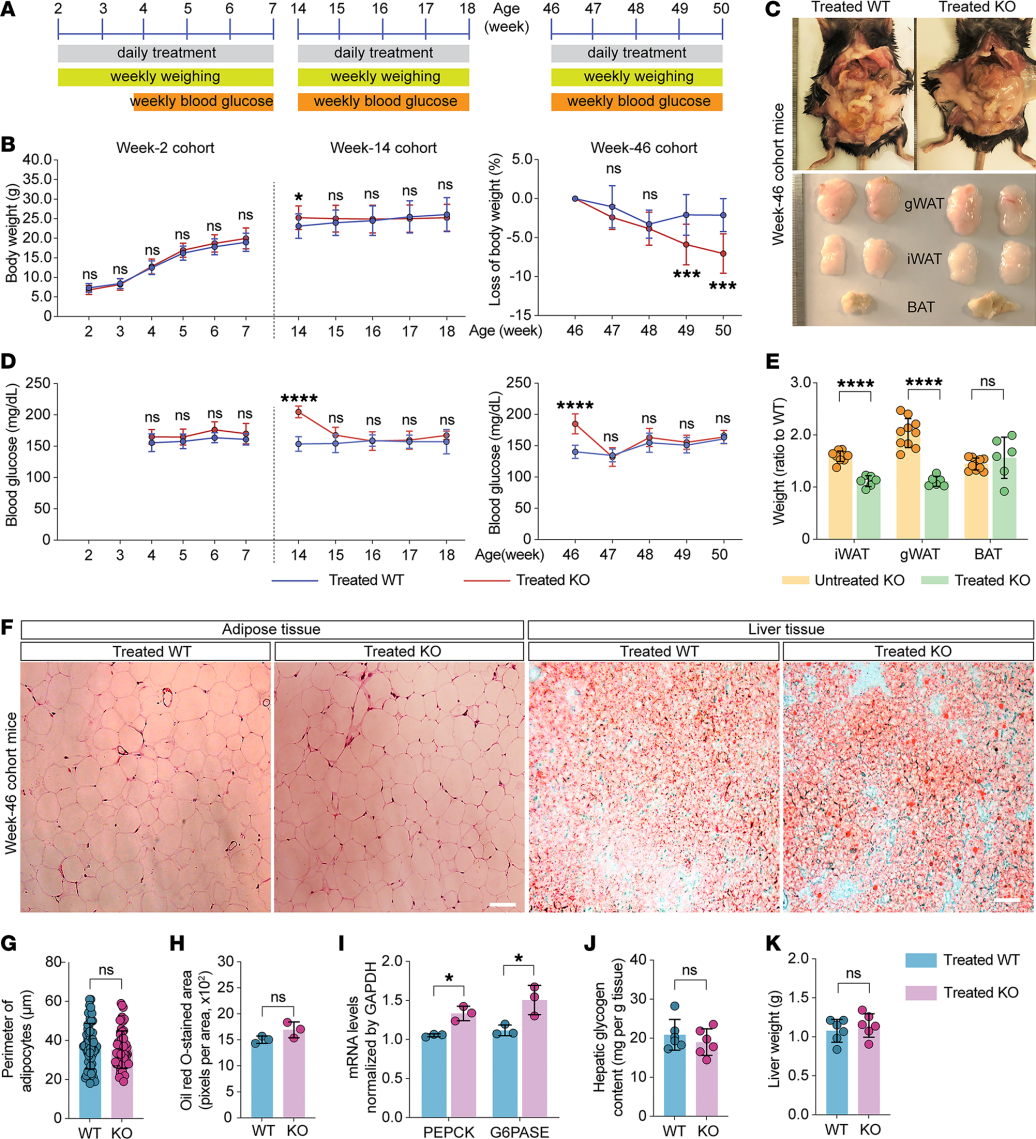
Chronic pharmacologic manipulation of dopamine transmission alleviates obesity and NAFLD in Trappc9-deficient mice. (**A**) Cohort assignment and experimental schedules. Week-2: *N* = 25 WT mice (8 male, 17 female), 16 KO mice (9 male, 7 female); week-14: *N* = 23 WT mice (9 male, 4 female), 12 KO mice (3 male, 9 female); week-46: *N* = 16 WT mice (8 male, 8 female), 18 KO mice (9 male, 9 female). After the treatment for 2 weeks, 2 male KO mice in the week-46 cohort were dead. (**B**) Blood glucose levels and (**C**) body weight (week-2 and week-14 cohorts) or body weight loss (week-46 cohort) and in WT and Trappc9-KO mice treated daily with SCH23390 and quinpirole for consecutive 6 (week-2 cohort) or 4 (week-14 and week-46 cohorts) weeks. Body weight and blood glucose levels from mice in each cohort at each time point were averaged and graphed. After the drug treatment, mice in the week-46 cohort were sacrificed for examining body adiposity (**D** and **E**), adipocyte size (**F** and **G**), lipid deposition in liver (**F** and **H**), PEPCK and G6PASE gene transcripts in liver (**I**), hepatic glycogen contents (**J**), and liver weight (**K**). The adipose tissues were dissected from the corresponding compartments (**D**) and weighed. Adipose tissue weight as percentage of body weight was used for calculating the ratio of Trappc9-KO adipose tissue weight to WT adipose tissue weight and graphed (**E**). (**F**) Images of hematoxylin and eosin–stained adipose tissue sections and Oil Red O-stained liver tissue sections. For densitometry, images captured from at least 3 sections from different compartments (adipose tissue) or lobes (liver) for each mouse were analyzed (*N* = 3 mice per genotype). Each symbol in bar graphs represents 1 mouse (**E** and **H**–**K**) or 1 cell (**G**). Data are mean ± SD. Two-tailed Student’s *t* test: *P < 0.05; ***P < 0.005; ****P < 0.001.

To challenge this idea, we first examined whether the treatment with SCH23390 and quinpirole mitigated hyperglycemia, hyperinsulinemia, and hyperprolactinemia in Trappc9-KO mice, as dopamine transmission modulates glucose homeostasis and inhibits prolactin and insulin secretion (40–43). A cohort of Trappc9-KO and WT mice were treated daily by intraperitoneal (i.p.) injection with a dose of SCH23390 and quinpirole. Levels of blood glucose were measured 24 hours after each dose. The treatment had no effect on blood glucose levels in WT mice but effectively lowered blood glucose of Trappc9-KO mice 5 days after the treatment; drug effects lasted for 2 weeks after the treatment was stopped (Supplemental Figure 3A). The SCH23390 and quinpirole treatment also effectively corrected the hyperinsulinemia and hyperprolactinemia in Trappc9-KO mice and resulted in corresponding changes in brain levels of cAMP (Supplemental Figure 3, B–D), indicating their actions in the brain. We then assessed whether chronic treatment combining SCH23390 and quinpirole prevented the development of obesity or NAFLD in Trappc9-KO mice. To this end, 3 cohorts of Trappc9-KO and WT mice, more specifically postnatal week 2, 14, and 46, were designated based on their age when the treatment was started (Figure 4A). The treatment was given daily to mice in the week-2 cohort for 6 weeks and to mice in both week-14 and week-46 cohorts for 4 weeks (Figure 4A). As expected, the levels of blood glucose in all 3 cohorts of Trappc9-KO mice were lowered to normal levels with the treatment (Figure 4B), suggesting that the disturbance of system glucose homeostasis in Trappc9-KO mice arises from altered dopamine transmission and is controllable with drugs acting on dopamine receptors.

### The SCH23390 and quinpirole treatment counteracts the abnormal gain of body weight in Trappc9-deficient mice.

Mice in the week-2 cohort are in the weaning period. While the body weight of WT mice in the week-14 cohort is relatively stable, Trappc9-KO mice at this age continue to gain body weight (19). Hence, the effect of the SCH2339 and quinpirole combined treatment on body weight in Trappc9-KO mice in both week-2 and week-14 cohorts was determined by directly comparing the body weight of Trappc9-KO mice with that of WT mice, whereas body weight loss was used as a measure of drug effects on body weight in the week-46 cohort. With treatment, Trappc9-KO and WT mice in the week-2 cohort exhibited a similar rate in gaining body weight, and Trappc9-KO mice in the week-14 cohort stopped gaining body weight (Figure 4C). The combined treatment caused Trappc9-KO mice in the week-46 cohort to lose more weight than their WT counterparts (Figure 4C). These data suggest that chronic pharmacologic manipulation of dopamine signaling mitigates the abnormal gain of body weight in Trappc9-deficient mice.

### The SCH23390 and quinpirole combined treatment alleviates lipid accumulation in adipose and liver tissues in Trappc9-deficient mice.

To examine the effect of the combined treatment on body adiposity, mice from the week-46 cohort were sacrificed after treatment. The adipose tissues from abdominal, gonadal, and inguinal compartments as well as the liver of each sacrificed mouse were harvested for examination. The abdominal fat deposition in Trappc9-KO mice was not overtly different from that in WT mice after the combined treatment for 4 weeks (Figure 4D). The iWAT and gWAT as well as BAT fat depots from drug-treated Trappc9-KO mice appeared similar in size (Figure 4D). A comparison of the ratio of Trappc9-KO adipose tissue weight (as percentage of body weight) to WT adipose tissue weight (as percentage of body weight) between drug-treated Trappc9-KO mice and untreated Trappc9-KO mice showed that the combined treatment resulted in a significant reduction of WATs (Figure 4E). Histological examination of WATs revealed that the size (perimeter) of adipocytes in drug-treated Trappc9-KO mice was no longer different from that in drug-treated WT mice (Figure 4, F and G), suggesting that lipid accumulation in adipose tissues was depressed with the combined treatment. In summary, the SCH23390 and quinpirole combined treatment effectively relieves body adiposity in Trappc9-KO mice.

Upon the combined treatment, Oil Red O staining in liver sections of Trappc9-KO mice was not different from that in liver sections of WT mice receiving the same treatment (Figure 4, F and H), indicating that the treatment attenuates lipid accumulation in the liver of Trappc9-KO mice. Quantitative PCR analysis found that the levels of transcripts for G6PASE and PEPCK in the liver of Trappc9-KO mice were reversed (Figure 4I). Concomitantly, the content of hepatic glycogen in drug-treated Trappc9-KO mice was lowered to normal levels (Figure 4J). These results suggest that the combined treatment restores glucose metabolism in the liver of Trappc9-KO mice. The weight of the liver of Trappc9-KO mice receded to the level of WT mice (Figure 4K). Together, our data suggest that chronic treatment with drugs acting on dopamine receptors blunts the development of obesity and NAFLD in Trappc9-deficient mice.

### Deficient stimulation of DRD2-containing neurons underlies the homeostatic disturbance systemic glucose in Trappc9-deficient mice.

**Figure 5:**
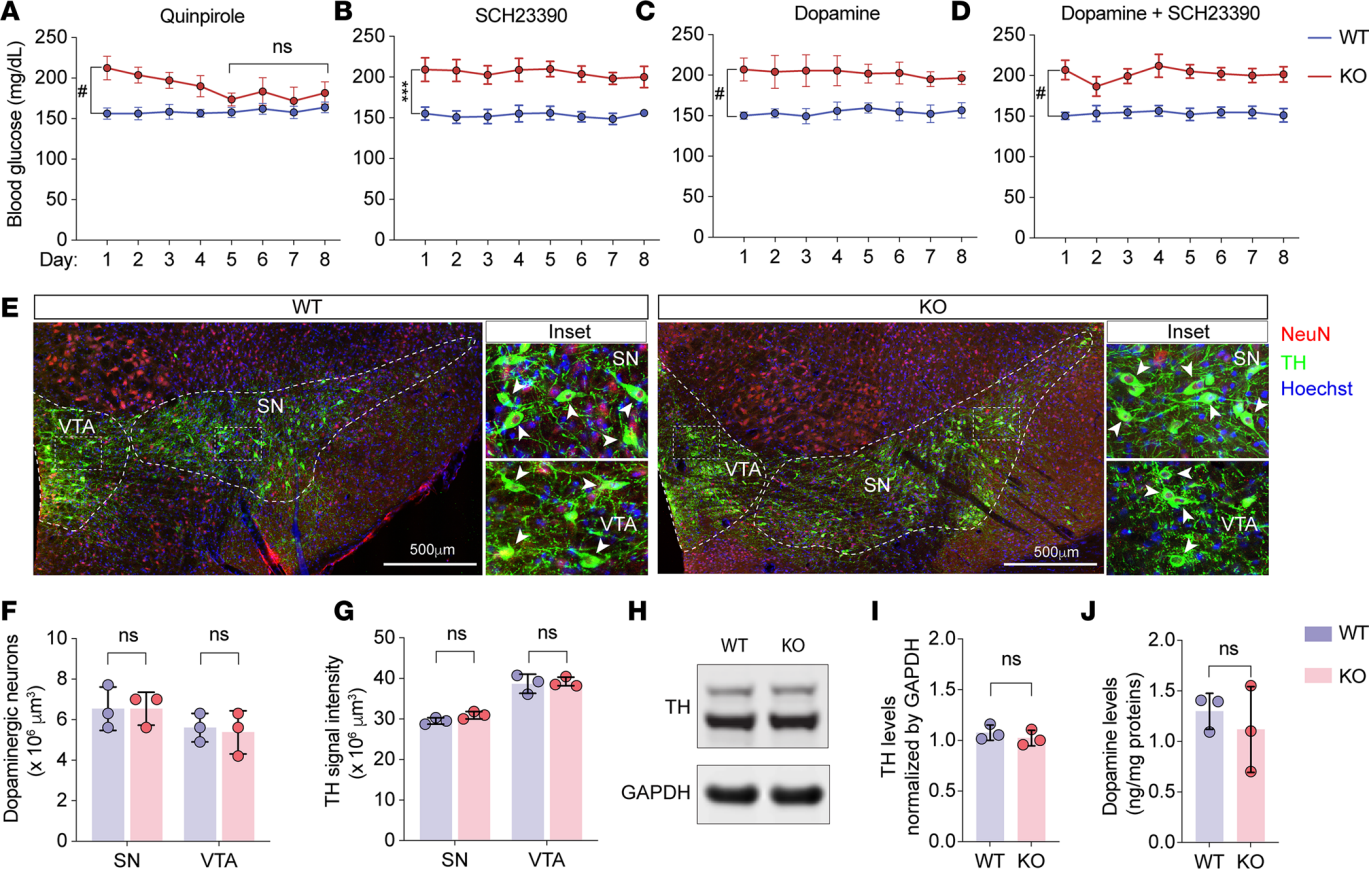
Trappc9 deficiency disrupts systemic glucose homeostasis by abating DRD2 stimulation without affecting dopamine synthesis in the brain. Comparisons of blood glucose levels in WT and Trappc9-KO mice receiving daily treatment, i.p., with quinpirole alone (**A**), with SCH23390 alone (**B**), with brain-impermeable dopamine (**C**), or with dopamine combined with SCH23390 (**D**) for 8 consecutive days. (**E**) Confocal images taken from the midbrain area of WT and Trappc9-KO mouse brain sections stained with antibodies for neuronal marker NeuN (red), dopaminergic neuronal marker TH (green), and DNA dyes (blue). Boxed regions within the VTA and SN, respectively, were enlarged as insets of original magnification, ×3.5. VTA, ventral tegmental area; SN, substantia nigra. Stereology counting of somata containing both NeuN and TH (**F**) and densitometric quantification of TH signals (**G**) within the indicated area closed with a contour in confocal images (**E**). Confocal images used for quantitative analyses were obtained from 3 consecutive brain sections from each brain (*N* = 3 mice per genotype). (**H**) Western blot analysis of whole brain lysates followed by densitometry of TH immunoreactive signals (**I**). (**J**) Comparison of dopamine levels in whole brain lysates of WT and Trappc9-KO mice. Pharmacological studies (**A**–**D**) were performed with 7 mice per genotype. Each symbol in bar graphs (**F**, **G**, **I**, and **J**) represents 1 animal. Data are mean ± SD. Two-tailed Student’s *t* test: ***P < 0.005; ^#^*P* < 0.0001.

Our above studies showed that the treatment combining SCH23390 and quinpirole restored the homeostasis of systemic glucose and counteracted obesity and NAFLD. To determine whether the disruption of systemic glucose homeostasis was primarily driven by altered dopamine transmission through DRD1 or DRD2 or both, a cohort of mice was treated with either quinpirole or SCH23390. Our results showed that quinpirole effectively reduced blood glucose of Trappc9-KO mice (Figure 5A), yet SCH23390 had a trivial effect on blood glucose levels in Trappc9-KO mice (Figure 5B), suggesting that deficient stimulation of DRD2 is the key contributor. As DRD2 is also expressed in peripheral tissues, e.g., the pancreas and adipose tissues (44–46), we determined whether the blood glucose–lowering effect of quinpirole in Trappc9-KO mice was achieved through its central or peripheral actions. To this end, we gave mice daily by i.p. injection a dose of dopamine, which is unable to pass through the blood-brain barrier and thus can act only in the peripheral tissues. The idea was that if quinpirole lowered blood glucose in Trappc9-KO mice through its peripheral actions, dopamine should also be effective. However, the level of blood glucose in Trappc9-KO mice did not decline upon daily treatment with dopamine over 8 days (Figure 5C), indicating that the disturbance of systemic glucose homeostasis most likely stems from deficient stimulation of DRD2 in the brain. To examine whether the suppression of DRD1 was necessary for dopamine to lower blood glucose in Trappc9-KO mice, mice were treated daily with SCH23390 combined with dopamine. Our data showed that SCH23390 combined with dopamine did not change blood glucose levels in Trappc9-KO mice (Figure 5D). Collectively, these data suggest that the disruption of systemic glucose homeostasis in Trappc9 KO is a consequence of deficient stimulation of DRD2 in the brain.

### Trappc9-deficient mice are normal in synthesizing dopamine but exhibit signs of impairments in dopamine secretion.

The effectiveness of SCH23390 and quinpirole in relieving obesity and NAFLD in Trappc9-KO mice indicates that when ligands are sufficiently available, DRD1- or DRD2-containing neurons as well as their effector cells in Trappc9-KO mice function normally. Hence, we examined whether there was a loss of capacity of synthesizing dopamine in Trappc9-KO mice. The dopaminergic neurons in the midbrain (the substantia nigra pars compacta and the ventral tegmental area) are the major sources of dopamine in the brain (46, 47). Histological and Western blot analyses showed that compared with WT mice, Trappc9 KO contained a normal cell density of dopaminergic neurons in the midbrain and expressed comparable levels of tyrosine hydroxylase (TH), the rate-limiting enzyme in dopamine synthesis (Figure 5, E–I). Consistently, similar levels of dopamine were detected in brain lysates of Trappc9-KO and WT mice (Figure 5J). These results suggest that Trappc9-KO mice synthesize dopamine normally in the brain.

**Figure 6:**
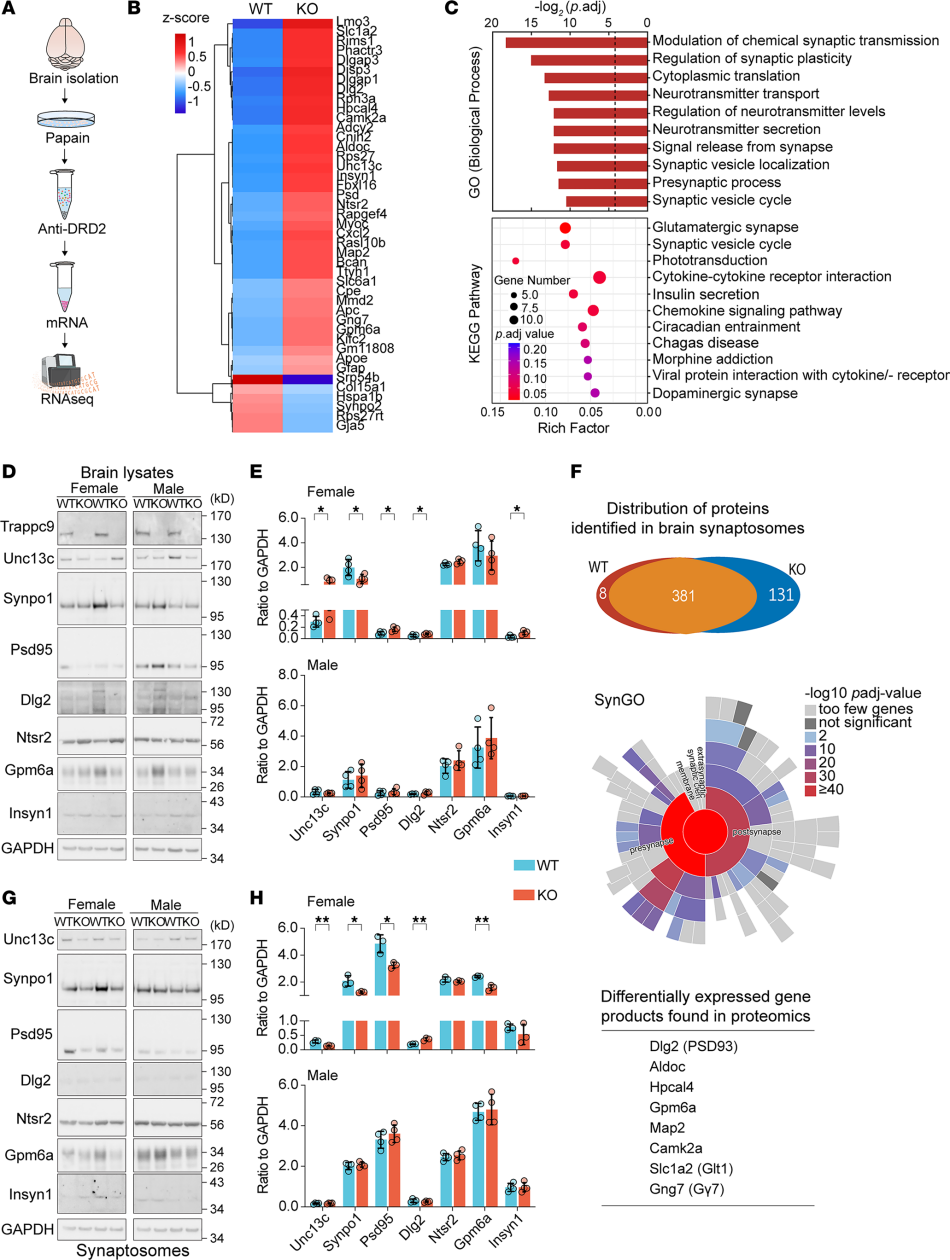
Transcriptomic and proteomic analyses reveal signs of impairments in neurotransmitter secretion in Trappc9-deficient mice. (**A**) Experimental schedule for RNA-Seq analysis of DRD2-containing cells from WT and Trappc9-KO mice (*N* = 3 mice per genotype). (**B**) Heatmap representation of the differentially expressed genes identified by RNA-Seq analysis of DRD2-containing cells. (**C**) GO function and KEGG pathway analyses of differentially expressed genes. Shown are top 10 GO biological processes and KEGG pathways, respectively. (**D**) Western blot analysis of gene products of some differentially expressed genes in brain lysates followed by densitometric quantification of the protein levels (**E**). (**F**) Overall distribution of proteins identified by proteomic analysis of brain synaptosomes (upper panel), Synaptic Gene Ontologies (SynGO) analysis of differentially expressed proteins with a change by at least 2-fold (middle panel), and summary of differentially expressed genes identified in both RNA-Seq and proteomic analyses (lower panel). (**G**) Western blot analysis of the levels of the chosen proteins as in **D** in brain synaptosomes of WT and Trappc9-KO mice followed by (**H**) densitometric quantification of the protein levels. Each symbol in bar graphs represents 1 mouse. Data are mean ± SD. Two-tailed Student’s *t* test: *P < 0.05; **P < 0.01.

The above studies suggest that deficient stimulation of DRD2 in the brain was a key contributor to the disruption of systemic glucose homeostasis in Trappc9-KO mice. To disclose potential molecular changes, DRD2-containing neurons were immunoisolated from Trappc9-KO mice and WT littermates and processed for RNA-Seq analysis (Figure 6A). DRD2 transcript was detected but not altered in Trappc9-KO mice (Supplemental Table 4). However, our analysis identified 6 downregulated and 37 upregulated protein-coding genes in DRD2-containing cells of Trappc9-KO mice (Figure 6B and Supplemental Table 4). GO and KEGG analyses implied alterations in neurotransmitter secretion and synapse formation in Trappc9-KO mice (Figure 6C and Supplemental Tables 5 and 6). Among the identified genes, Unc13c (Munc13-3) and Rims1 (RIM) are known to manage dopamine secretion, whereas Gpm6a, Synpo, Insyn1, and Dlg2 (Psd93) are involved in forming and/or stabilizing synapses. NtsR2 was also the top hit of the upregulated genes in DRD2-containing neurons of Trappc9-KO mice. Given their functions in synaptic transmission, their protein levels were examined by Western blot analysis. The results showed that the levels of Unc13c, Dlg2, and Insyn1 were elevated whereas the level of Synpo1 was reduced in brain lysates of female Trappc9-KO mice relative to their levels in female WT counterparts (Figure 6, D and E). However, the levels of these proteins in male KO mice were comparable to those in male WT mice (Figure 6, D and E). It is not clear whether this divergence is related to the sex difference in time of onset of obesity and severity of phenotypes between female and male Trappc9-KO mice (19, 22).

To further demonstrate synaptic perturbation in Trappc9-KO mice, synaptosomes were prepared from fresh brain tissues of 3- to 4-month-old WT and Trappc9-KO mice. Quantitative proteomics analysis identified 8 proteins only present in WT mouse synaptosomes and 131 only in synaptosomes of Trappc9-KO mice (Figure 6F and Supplemental Table 7). SynGO analysis of the differentially expressed proteins with a change by at least 2-fold showed that both presynaptic and postsynaptic proteins were altered in Trappc9-KO mice (Figure 6F and Supplemental Table 8). It is worth noting that 8 genes/proteins were identified in both RNA-Seq and proteomic analyses (Figure 6F). Western blot analysis of the above set of proteins revealed that their levels were diminished in brain synaptosomes from female Trappc9-KO mice and unchanged in synaptosomes from male Trappc9-KO mice (Figure 6, G and H). Collectively, these data suggest that the deficiency of Trappc9 indeed constrains synapse formation.

### Trappc9 deficiency reduces the abundance of dopamine axonal varicosities.

**Figure 7:**
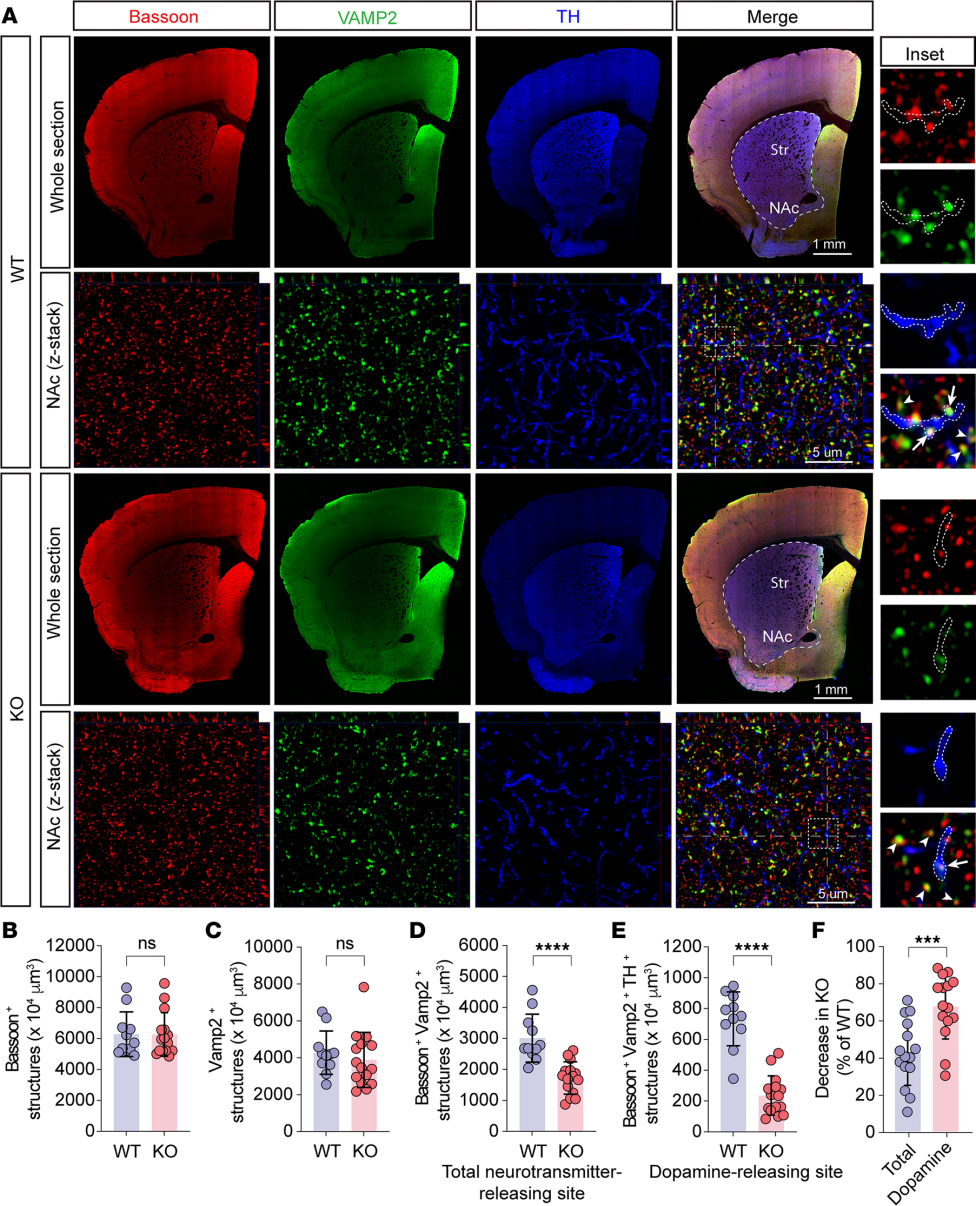
The abundance of dopamine release sites is diminished in the brain of Trappc9-deficient mice. A series of 3 consecutive sections per brain (*N* = 3 mice/genotype) were labeled with antibodies for Bassoon, Vamp2, and TH to detect dopamine release sites. (**A**) Shown are low-magnification images of a brain section and high-magnification *Z*-stack images taken within the NAc. Boxed regions in the high-magnification merged *Z*-stack images were enlarged as insets of original magnification, ×3.23. *Z*-stack images were used for counting the frequency of structures positive for Bassoon (**B**), Vamp2 (**C**), or both Bassoon and Vamp2 (**D**) and the frequency of those Bassoon^+^ and Vamp2^+^ structures in TH^+^ processes (**E**) with stereology methods. The percentage of reduction of structures positive for both Bassoon and Vamp2 (total release sites) and the percentage of reduction of Bassoon^+^ and Vamp2^+^ structures within TH^+^ processes (dopamine release sites) were calculated and graphed (**F**). In enlarged merged images (inset), arrows point to dopamine release sites, whereas arrowheads indicate the release sites of other neurotransmitters. Contours indicate TH^+^ axonal segments. Each symbol in bar graphs represents 1 mouse. Data are mean ± SD. Two-tailed Student’s *t* test: ***P < 0.005; ****P < 0.0001. Str, striatum.

Having found clues of impairments in neurotransmitter secretion and synapse formation, we examined whether dopamine synapses were altered in Trappc9-KO mice. In projection areas, e.g., the striatum, dopaminergic neurons release dopamine at their axonal varicosities, which synapse onto dendrites or dendritic spines of dopamine-receptive neurons (48, 49). In this regard, dopamine synapse alterations can be reflected from a change in dopamine-secreting axonal varicosities. The abrogation of Trappc9 in mice diminishes dendritic spines (21) and alters the density of dopamine-receptive neurons (19). As dopamine neurotransmission in the nucleus accumbens (NAc) is involved in body weight control and regulates systemic glucose homeostasis (25, 42, 43, 50–52), we focused our analysis on dopamine axonal varicosities in the NAc. To this end, brain sections containing the striatum were colabeled with antibodies for TH, active zone scaffold protein Bassoon, and synaptic vesicle protein Vamp2 to identify dopamine-secreting axonal varicosities (Figure 7A). While there was no difference in the frequency of structures positive for Bassoon or for Vamp2 between WT and Trappc9-KO mice (Figure 7, B and C), the structures containing both Bassoon and Vamp2 (total neurotransmitter release sites) were less abundant in the striatum of Trappc9-KO than in the striatum of WT mice (Figure 7D), suggesting that Trappc9 deficiency reduces the abundance of synapses. Not surprisingly, the frequency of structures positive for both Bassoon and Vamp2 in TH-positive processes was reduced in Trappc9-KO mice (Figure 7E). Further analysis revealed that the magnitude of decrease of dopamine-secreting varicosities was significantly greater than that of total neurotransmitter-releasing sites (Figure 7F), suggestive of differential vulnerability of dopamine synapse to the loss of Trappc9.

## Discussion

Human studies have suggested Trappc9 to be a risk factor for obesity and NAFLD, but the relevant mechanism is not clear. In this study, we found that the ablation of Trappc9 resulted in disruption of systemic glucose homeostasis, which was associated with hyperinsulinemia, hyperleptinemia, hyperprolactinemia, dyslipidemia, adipocyte hypertrophy, and glucose metabolism reprogramming and lipid accumulation in the liver. Transcriptomic analysis of ASCs implied global metabolic changes and the involvement of dopamine transmission. Chronic pharmacologic manipulation of dopamine transmission restored systemic glucose homeostasis and relieved obesity and NAFLD in Trappc9-KO mice. The disruption of systemic glucose homeostasis in Trappc9-KO mice was attenuated upon pharmacologically stimulating DRD2 alone. Transcriptomic and proteomic analyses pointed to impairments in neurotransmitter secretion in Trappc9-KO mice. Consistently, Trappc9-KO mice synthesized dopamine normally in the brain, but their dopamine-secreting neurons had a reduced abundance of structures for releasing dopamine in the nucleus accumbens. Our study suggests that Trappc9 loss of function causes obesity and NAFLD by perturbing dopaminergic synapse formation.

**Figure 8:**
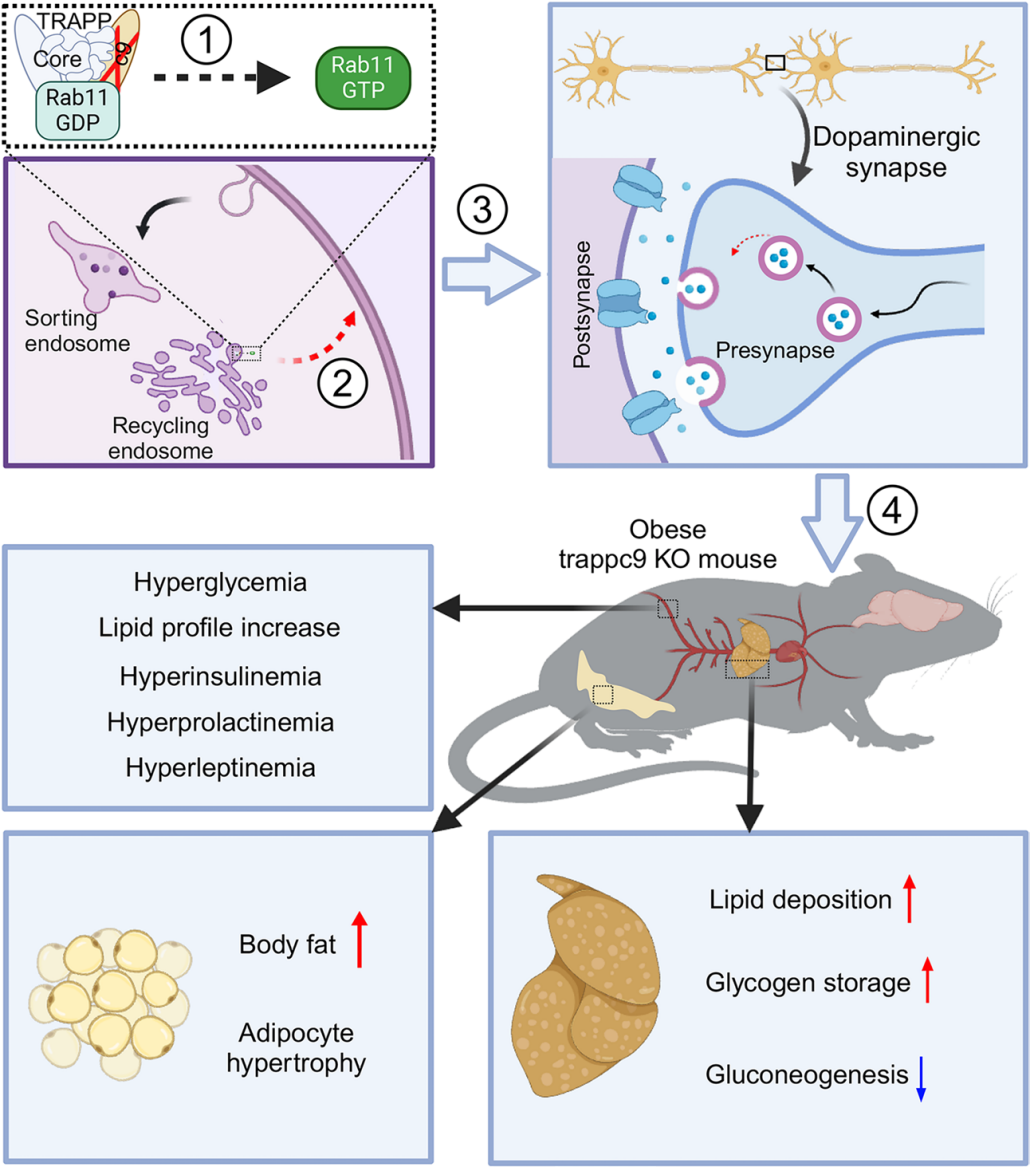
Schematic representation of obesity onset induced by the deficiency of Trappc9. In this simplified model, the impaired activation of Rab11 is the driving force. 1) The loss of Trappc9 abolishes the formation of fully functional TRAPPII for activating Rab11 and results in Rab11 functional decline; 2) a reduction in Rab11 function impedes endocytic recycling of internalized plasma membrane lipids and proteins from the recycling endosome to the cell surface, thereby disturbing cell functions; 3) chronic impairment of Rab11-based trafficking leads to a decline in the formation of dopaminergic synapses, thereby restraining dopamine transmission; 4) dopamine neurotransmission alteration disrupts systemic glucose homeostasis and triggers a cascade of metabolic changes, including excessive secretion of insulin, prolactin and leptin, and lipid deposition in adipose and liver tissues, and eventually leads to the onset of obesity and NAFLD. The scheme was drawn with BioRender (www.biorender.com).

Along with prior findings that Trappc9 ablation dampens Rab11 activation and reduces dendritic spines (19, 21, 53), our studies support a model for the onset of obesity triggered by Trappc9 deficiency. In this simplified model (Figure 8), impaired activation of Rab11 is the driving force. 1) A loss of Trappc9 abolishes the formation of fully functional TRAPPII for activating Rab11 and results in Rab11 functional decline; 2) a reduction in Rab11 function impedes the recycling of internalized plasma membrane lipids and proteins from recycling endosomes to cell surfaces, thereby disturbing cell functions; 3) chronic impairment of Rab11-regulated trafficking diminishes the abundance of dopaminergic synapses; and 4) altered dopamine transmission disrupts systemic glucose homeostasis, thereby triggering a cascade of metabolic changes, including excessive secretion of insulin, prolactin and leptin, and lipid deposition in adipose and liver tissues, eventually leading to the onset of obesity and NAFLD.

While the Trappc9 syndrome is extremely rare, Trappc9 variations may contribute significantly to the prevalence of obesity based on the following features of Trappc9 pathogenic mutations. First, they are highly diverse, which range from nonsense or missense point mutations to indels to large DNA fragment deletions. Second, they are not clustered within certain “hot spots” but scattered throughout the gene and occur in exons, introns, and splicing sites, suggesting that they are not restricted to persons of certain races or geographical regions. Third, Trappc9 mutations are inheritable from parents with no family history of consanguineous marriage (9, 54) and even de novo (55, 56). Moreover, abnormal epigenetic modification of the Trappc9 gene is associated with obesity in humans (12–14). It is not clear how common Trappc9 mutations are in the general population. Over 4,000 Trappc9 variants are found in the gnomAD V4.0 data set (https://gnomad.broadinstitute.org/gene/ENSG00000167632?dataset=gnomad_r4), in which several known disease-causing mutations were not included.

In mice, Trappc9 is imprinted specifically in the brain with a maternal allele–biased expression (~70%) and shows an equal biallelic expression in peripheral tissues (20, 57). Trappc9 mice with only the maternal allele mutated appear overweight, whereas mice with only the paternal allele mutated are normal (20), indicating that obesity development in Trappc9-KO mice involves a loss of function mainly in the brain. Prior studies reveal that the abrogation of Trappc9 diminishes DRD2-containing neurons and increases DRD1-containing neurons in the striatum; acute treatment combining quinpirole and SCH23390 to stimulate DRD2 and repress DRD1 effectively attenuates defects associated with multiple behaviors (19). In this study, we found that chronic treatment combining quinpirole and SCH2330 counteracted abnormal body weight gain and alleviated lipid accumulation in adipose and liver tissues in Trappc9-KO mice. Quinpirole alone was sufficient to restore blood glucose homeostasis in Trappc9-KO mice, but peripheral administration of SCH23390 alone or brain-impermeable dopamine or their combination had no such effect. These findings suggest that the development of obesity in Trappc9-KO mice is related to an improper function of the dopamine system in the brain.

Dopamine transmission depends on the adequacy of dopamine in extracellular spaces and dopamine receptors on the cell surface. On the cell surface of striatal neurons, levels of DRD1 are increased whereas levels of DRD2 are decreased in Trappc9-KO mice (19). If dopamine were sufficiently accessible, such changes in DRD1/2 levels on neuronal surfaces should render Trappc9-KO mice hyperactive based on the classical view of motor control (58, 59). On the contrary, they are hypoactive (19). The hypoactivity of Trappc9-KO mice is unlikely to stem from insufficient excitatory inputs because animal immobility appears in Trappc9-KO mice as quickly as in WT mice, a few minutes after administrating quinpirole or SCH23390 (19). As dopamine can bind to its receptors only when it is released into extracellular spaces, altered dopamine transmission in Trappc9-KO mice most likely results from defective secretion of dopamine. In projection areas, e.g., the striatum, dopaminergic neurons secrete dopamine at axonal varicosities, which synapse onto dendrites or dendritic spines of their innervated neurons (48, 49). Our studies showed that Trappc9-KO mice synthesized normal levels of dopamine in the brain, but their dopaminergic neurons had a lower abundance of dopamine release sites on axons in the NAc. This could be related to alterations in the expression of proteins involved in organizing neurotransmitter-secreting structures in dopaminergic neurons. Alternatively, the decrease of dopamine-releasing structures is a compensatory response to changes in dendritic spines, which are reduced in Trappc9-KO mice (21).

Dopamine impacts systemic glucose homeostasis and body weight through both central and peripheral actions. Centrally, dopamine regulates food intake, thermogenesis, and hormone secretion in the pituitary gland (28), whereas peripherally, it modulates the secretion of insulin and adipokines as well as insulin-stimulated uptake of glucose (41, 60–62). In this regard, the effect of SCH23390 and quinpirole on glucose metabolism and obesity onset in Trappc9-KO mice might be achieved through actions in peripheral tissues. If so, peripheral administration of dopamine combined with SCH23390 should mimic the combination of SCH23390 and quinpirole, because peripherally administrated dopamine is unable to cross the blood-brain barrier and can act only in peripheral tissues. However, neither dopamine nor SCH23390 nor their combination was able to lower blood glucose levels in Trappc9-KO mice. On the other hand, quinpirole alone effectively downgraded blood glucose in Trappc9-KO mice. Hence, deficient stimulation of DRD2 in the brain is a key contributor.

ASCs are key regulators of adipose tissue remodeling by giving rise to adipocytes and maintaining immunological homeostasis in adipose tissues (33, 35). ASC dysfunction and obesity are intertwined to magnify the development of obesity and comorbidities (35). Trappc9-KO ASCs have signs of impairments associated with multiple cellular pathways and contain abnormally large lipid droplets after being induced to adipogenic differentiation (23). Abnormal accumulation of lipid droplets is also seen in hippocampal neurons cultured from Trappc9-KO mice (22). Consistent with these findings of lipid droplet accumulation in Trappc9-KO cells, RNA-Seq analysis revealed changes in the expression of genes relevant to lipid metabolism in ASCs isolated from Trappc9-KO mice. DRD1 and DRD2 transcripts were detected in ASCs, but their levels were not altered in Trappc9-KO ASCs. It is not clear whether dopamine signaling is altered in Trappc9-KO ASCs, or whether dopamine signaling is related to the changes found in Trappc9-KO ASCs.

Exactly how Trappc9 loss of function disturbs metabolism is not clear. Trappc9 is a subunit specific for TRAPPII, which tethers transport vesicles and activates Rab proteins (63, 64). Trappc9 regulates lipid droplet homeostasis by activating and recruiting Rab18 onto surfaces of lipid droplets (64). Trappc9-KO ASCs express Rab18 at higher levels relative to WT ASCs (23). Disease-causing mutations result in the absence of the Trappc9 protein, making it impossible to assemble functional TRAPPII for activating Rab proteins (19, 65). Hence, metabolic disturbance and obesity onset in Trappc9-KO mice most likely involve dysfunction of TRAPPII. In addition to Trappc9, several other components of TRAPPII are also linked to human diseases, which include Trappc2, Trappc2l, Trappc4, Trapp6A/B, and Trappc10. In TRAPPII, Trappc9 interacts with Trappc10 to form a subcomplex and with Trappc2 to associate the TRAPP core (53, 65). The deficiency of Trappc9 and Trappc10 mutually depletes each other in mice (19, 66). Like Trappc9-KO mice, trappc10-deficient mice develop obesity postnatally (66). However, patients bearing mutations of Trappc2, Trappc2l, Trappc4, Trappc6A/B, or Trappc10 do not develop obesity as patients with Trappc9 mutations do. In this regard, the development of obesity caused by Trappc9 mutations may involve a function independent of its association with TRAPPII.

Trappc9 has been shown to potentiate the activation of NF-κB (67). Yet, no sign of defective activation of NF-κB was found in 2 different lines of Trappc9-KO mice (19, 21). As deficient NF-κB activation in fibroblasts of patients with Trappc9 mutations is seen upon stimulation with tumor necrosis factor-α (3), NF-κB activation may be altered in Trappc9-KO mice under induction conditions. Based on these observations, Trappc9 deficiency should hamper obesity development, as blunting NF-κB activation abates the development of obesity (68, 69). On the contrary, Trappc9 deficiency results in obesity in both humans and mice. Alternatively, NF-κB activation might be enhanced in Trappc9-KO mice under stimulation conditions. If so, NF-κB activation in the Trappc9-KO setting should occur through another mechanism. DRD2-mediated signaling through β-arrestin 2, Akt, and protein phosphatase 2A represses NF-κB activation (70). Future studies are needed to challenge this idea.

### Conclusions, limitations, and perspectives.

Collectively, this study shows that Trappc9 deficiency causes increased body weight, and this augmented body weight gain leads to disruptions of glucose metabolism and fatty liver, thus establishing the Trappc9 mouse line as a tool for studying obesity and NAFLD, and provides a potential treatment for obesity and NAFLD. However, this study has limitations. First of all, our study only examined the contribution of dopamine transmission to the development of obesity in Trappc9-KO mice. Other neurotransmitters or neuromodulators, neurotensin in particular, may also be affected and in operation, as implicated by findings from RNA-Seq analysis of ASCs as well as of DRD2-containing neurons, proteomic profiling of brain synaptosomes, and histological examination of neurotransmitter-releasing sites. As the neurotensin system plays a pivotal role in body weight control and regulates dopamine secretion, future studies are needed to examine whether the function of the neurotensin system is altered in Trappc9-KO mice. Second, Trappc9 is expressed in virtually all major types of cells in the brain. A large body of evidence indicates that astrogliosis contributes significantly to the pathogenesis of obesity. In the brain of Trappc9-KO mice, there exists astrogliosis. Astrocytes also express DRD2. Hence, obesity onset in Trappc9-KO mice and the beneficial effects of quinpirole may involve functional change associated with astrocytes. While we suggest altered dopamine transmission in the brain to be a key contributor, it is not clear to us in which brain region(s) dopamine transmission alteration plays a dominant role in the onset of obesity in Trappc9-KO mice. Prior studies and the results in this study suggest ASC dysfunction in Trappc9-KO mice. It is also necessary for future studies to determine what role(s) ASC dysfunction plays in the development of obesity in Trappc9-KO mice. As discussed above, the molecular pathway by which Trappc9 deficiency disturbs metabolism still needs to be clarified.

## Methods

### Sex as a biological variable.

Although female Trappc9-KO mice develop more robust and broader behavior defects than male KO mice (19), both female and male Trappc9-KO mice appear overweight. Studies in this report involved mice of both sexes.

### Animals.

Animals were housed in male and female groups and maintained in a pathogen-free environment under regular 12-hour light/12-hour dark cycle with food and water ad libitum. All mice were genotyped by PCR as described (19). Experimental procedures were carried out following the NIH *Guide for the Care and Use of Laboratory Animals* (National Academies Press, 2011).

### Serum biochemistry profile.

Blood samples were collected from WT and Trappc9-KO mice and centrifuged at 4°C at 1,000*g* for 12 minutes. The resulting plasmas were subjected to measuring levels of triglyceride, cholesterol, high-density lipoprotein, low-density lipoprotein, urea, creatinine, AST, and ALT (Servicebio company).

### Glucose and insulin tolerance tests.

Levels of blood glucose were measured with a glucometer (Accu-Check Active, Roche) following the supplier’s procedures. The first drop of blood was discarded, and the second drop was placed on a test strip, which was then inserted into the glucometer. For the GTT, mice were fasted overnight (14 hours) with access only to drinking water throughout the test. The body weight and fasting blood glucose levels of each mouse were measured and recorded before starting the GTT. Each mouse was given by i.p. injection 2 g glucose per kilogram body weight, which was prepared with 20% glucose (MilliporeSigma). Levels of blood glucose were measured at 15, 30, 60, and 120 minutes after concentrated glucose was injected. Blood loss was minimized by slightly pressing and cleaning the incision area with 75% alcohol swabs after each measurement. At the end of the GTT, each mouse was provided with abundant food and water and observed for 1–2 days for any odd behavior. For the ITT, mice were fasted for 6 hours between 7 am and 1 pm as above. After fasting, the body weight and levels of blood glucose of each mouse were measured. Each mouse was the given by i.p. injection 0.75 IU insulin per kilogram body weight. Levels of blood glucose were measured at 15, 30, 60, and 120 minutes after insulin was given. After the ITT was completed, mice were transferred to clean cages with free access to food and water and monitored for 2–4 days for abnormal behaviors.

### ELISA.

Plasma levels of leptin, prolactin, and insulin were measured by ELISA with reagents obtained from Ray Biotech, CusaBio, and Crystal Chem, respectively, according to the reagent suppliers’ instructions. The optical density at 450 nm of each sample was measured in a multifunctional microplate reader (Biotech, Synergy LX). The absorbance for each set of standards was measured. The standard curve was drawn using the curve expert and GraphPad Prism, and the best-fit curve was drawn through these points.

### Pharmacological treatment.

To assess the effect of the combined treatment with SCH23390 and quinpirole on blood glucose, a cohort of WT and Trappc9-KO mice at age 3–4 months were given daily i.p. a dose of both SCH23390 at 0.1 mg/kg (Tocris Bioscience, catalog 0925) and quinpirole at 1 mg/kg (Tocris Bioscience, catalog 1061). Levels of blood glucose were measured 24 hours after each dose. Another cohort of mice at age 3–4 months was used for determining whether the blood glucose–lowering effect originated from SCH23390, or quinpirole, or both and for determining the effect of dopamine at 0.1 mg/kg (dopamine hydrochloride, MilliporeSigma, catalog EY1491) in lowering blood glucose levels. For evaluating the effect of the chronic treatment combining SCH23390 (0.1 mg/kg) and quinpirole (1 mg/kg) on obesity and NAFLD, 3 cohorts of WT and Trappc9-KO mice were devised based on their postnatal age when the treatment was started: 2 weeks, 14 weeks, and 46 weeks. The drugs were administered by i.p. injection every day. Body weight and blood glucose levels were measured weekly.

### Histological examination of adipose tissues.

Abdominal white and interscapular brown fat tissues were surgically removed from each deeply anesthetized mouse, transferred to a glass vial containing 4% paraformaldehyde, and fixed overnight at 4°C. Fixed adipose tissues were washed several times in phosphate-buffered saline (PBS), embedded in paraffin, and cut into 5 μm sections, which were collected onto glass slides. The sections were then stained in the hematoxylin solution for 5 minutes. After washes in distilled water, the sections on slides were soaked in the eosin reagent for 1–2 minutes and then rinsed in distilled water. The sections were dehydrated sequentially in 75%, 80%, 90%, 95%, and 100% alcohol, 2 minutes in each, and then cleansed twice in xylene, each for 2 minutes. After being dried, the sections were coverslipped with mounting media and examined under an Olympus BX53 microscope. Digital images were taken from at least 3 randomly chosen visual fields for each section. The perimeter of adipocytes and the number of adipocytes per area were analyzed with NIH ImageJ/Fuji software.

### Oil Red O staining of liver tissues.

Fresh liver tissues were surgically removed from deeply anesthetized mice, washed multiple times with PBS, and embedded in OCT media. A series of 15 μm sections were cut on a cryostat (Leica). The sections were directly mounted onto glass slides and stained with 0.5% of Oil Red O solution for 10 minutes. The sections were then rinsed with running distilled water for 15 minutes, dried at room temperature, and coverslipped with water-soluble mounting media (MilliporeSigma). Digital images were captured from at least 3 randomly chosen visual fields using the Olympus BX53 microscopy imaging system with the same settings for each section. Oil Red O–stained areas were quantified with NIH ImageJ/Fuji software. Data were expressed as mean ± SD pixels per area.

### Hepatic glycogen measurement.

Hepatic glycogen was measured according to manufacturer’s instructions (SolarBio). In brief, 100 mg of liver tissues were homogenized in 0.75 mL of extraction solutions (reagent I). The homogenates were boiled in a water bath for 20 minutes and gently mixed every 5 minutes. After being cooled down to room temperature, the samples were transferred to a fresh tube containing 5 mL distilled water, mixed thoroughly, and centrifuged at 8,000*g* for 10 minutes. The resulting supernatants were transferred to a new tube. Subsequently, 60 μL from each sample was transferred into a well of a 96-well plate and mixed with 40 μL of reagent II containing mainly Anthrone reagent. The absorbance at 620 nm was recorded in a microplate reader (Biotech, Synergy LX). Glycogen content was expressed as mg/g liver tissue.

### RNA-Seq analysis.

ASCs were isolated as previously described (23). Briefly, abdominal fat pads were collected aseptically from 3 WT and 3 Trappc9-KO mice aged 3–4 weeks. Tissues were washed 3 times in Hank’s balanced salt solution, minced into small pieces, and then treated with 1 mg/mL type I collagenase (Yeasen Biotechnology) at 37°C with gentle mixing at 120 rpm. Enzymatic digestions were terminated by adding 10 mL of α-MEM containing 10% fetal bovine serum (FBS), 1× l-glutamine, and 1× penicillin/ streptomycin. The digestion mixtures were filtered through a 70 μm cell strainer (Thermo Fisher Scientific), and the filtrates were centrifuged at 200*g* for 5 minutes. The cell pellets were washed once in PBS and resuspended in 1 mL TRIzol reagents (Invitrogen) for RNA-Seq analysis.

DRD2-containing neurons were immunomagnetically isolated from both WT and Trappc9-KO mice at age 3–4 weeks. In brief, brain tissue was minced into small pieces and treated with 30 U/mL papain at 37°C for 20 minutes in neurobasal medium supplemented with 1% penicillin-streptomycin, 1% l-glutamine, 2% B27, and 2,500 U DNase I. The digestion was terminated by adding 1 mL FBS. The digested tissues were gently dissociated by pipetting through a blue tip and subsequently passed through 70 μm and 40 μm cell strainers (Thermo Fisher Scientific). Cells in the flow-throughs were collected by a centrifugation at 200*g* for 10 minutes and washed once with neurobasal medium with 10% FBS and all supplements as above. Cells were resuspended in PBS (pH 7.2) containing 0.5% bovine serum albumin, 0.5 mM EDTA, 5 μg/mL insulin, and 1 g/L glucose and incubated with DRD2-specific antibodies precoupled on magnetic beads at 4°C for 15 minutes. Cells on beads were washed once in the above PBS buffers, separated on a magnetic grate, and resuspended in 1 mL TRIzol reagents for RNA-Seq analysis.

RNA-Seq and data processing were performed at Novogene. Libraries were prepared using oligo(dT)-attached magnetic beads. Sequencing was performed on the Illumina platform. Raw data underwent thorough quality control analyses to remove adapters and low-quality reads. The processed reads were aligned to mouse genome (GRCm39 assembly), and gene expression was quantified via fragments per kilobase of transcript per million mapped reads. Differential gene expression was modeled in the DESeq2 R package (1.20.0) or edgeR R package (3.22.5). The resulting *P* values were adjusted using the Benjamini-Hochberg method. Adjusted *P* value of 0.05 and 2-fold changes were set as the threshold for significantly differential expression.

GO enrichment analysis of differentially expressed genes was implemented by the clusterProfiler R package (3.8.1), in which gene length bias was corrected. GO terms with Benjamini-Hochberg–adjusted *P* < 0.05 were considered significantly enriched. The clusterProfiler R package (3.8.1) was applied to test the statistical enrichment of differentially expressed genes in KEGG pathways. Protein-protein interaction networks of neurotensin receptors and dopamine receptors were obtained from the STRING database (https://www.string-db.org).

### Synaptosome preparation and proteomic analysis.

Synaptosomes were isolated from fresh brain tissues as previously described (71). Briefly, WT and Trappc9-KO mice aged at 4–5 months were anesthetized. The brain was removed and homogenized with a dounce homogenizer on ice in 7 mL freshly prepared homogenization buffer (0.32 M sucrose, 1 mM EDTA, 10 mM DTT, protease inhibitor). Homogenates were centrifuged at 4°C at 1,000*g* for 10 minutes. The resulting supernatants were layered onto 1.2 M sucrose in 14 × 89 mm Ultra-Clear centrifuge tubes (Beckman). The gradients were centrifuged at 4°C at 160,000*g* for 35 minutes (SW41 rotor) in a Beckman XPN-100 Ultracentrifuge. On the gradients, synaptosomes were visible as a cloudy band at the interface between 0.32 M and 1.2 M sucrose and collected. Protein concentrations in synaptosomes were measured.

Label-free quantitative proteomics analysis was conducted as previously described (72). In brief, synaptosomes (10 μg proteins) from each brain were mixed with an equal volume of acetone precooled to –20°C. After incubation for 60 minutes at –20°C, the samples were centrifuged at 4°C at 14,000*g* for 10 minutes. The pellets were dried in air for 30 minutes and subjected to mass spectrometry analysis at the Beth Israel Deaconess Medical Center Mass Spectrometry Core facility (https://www.bidmc.org/research/core-facilities/mass-spectrometry-proteomics-metabolomics-core). The LC-MS/MS spectra of peptides were analyzed using the Mascot Version 2.7.0 (Matrix Science) by searching the reversed and concatenated mouse protein database (Mouse_20221214.fasta) with a parent ion tolerance of 18 parts per million and fragment ion tolerance of 0.05 Da. Raw data of total spectrum count were imported into Scaffold (v5.2.2) software (Proteome Software) with a peptide threshold of ~50%, a protein threshold of ~95%, and at least 2 peptides. Further statistical analysis was performed using RStudio (v4.3.1). The raw data were adjusted by adding a value of 1 to all entries to account for 0 counts. Data processing was performed in R studio (v4.3.1) utilizing the limma (v3.58.1) package for linear modeling and differential expression analysis. Differential expression of a protein was assessed using an empirical Bayes method. A *P* value of 0.05 and a 2-fold change were set as the threshold for determining differential expression. GO and KEGG analyses were done as above in RNA-Seq analysis. The synapse-specific SynGO database was used to retrieve significantly enriched terms describing cellular components.

### SDS-PAGE and Western blot analysis.

SDS-PAGE and Western blot analysis were carried out as standard procedures. Primary antibodies used for Western blot analysis included Trappc9 (1:500; Abcam, ab104041), TH (1:5,000, Abcam, ab137869), Unc13-3 (1:1,000, Synaptic Systems, 126303), Synpo1 (1:2,000, Novus Biologicals, NBP2-39100), PSD95 (1:1,000, Cell Signaling Technology, 2507S), Dlg2 (1:200, Abcam, ab97290), NstR2 (1:5,000, Novus Biologicals, NB-100-56472SS), Gpm6a (1:5,000, Synaptic Systems, 238003), Insyn1 (1:200, MyBioSource, MBS3218282), GAPDH (1:10,000, EMD Millipore, MAB374), and β-actin (1:4,000, MilliporeSigma, A5441). Peroxidase AffiniPure secondary antibodies (Jackson ImmunoResearch, 715-035-151 and 711-035-152) were diluted at 1:10,000 in PBS containing 2% bovine serum albumin (MilliporeSigma). Blots were developed using Pierce enhanced chemiluminescence (Thermo Fisher Scientific) and imaged with the ChemiDoc MP Imaging System (Bio-Rad) or with a charge-coupled device camera (Alpha Innotech). Densitometry was conducted using the NIH ImageJ/Fiji software. Background signals were removed. Signal intensities for each protein of interest were normalized with those of GAPDH in the corresponding samples.

### Immunofluorescence microscopy and image analysis.

Mice aged 3 to 5 months were deeply anesthetized and transcardially perfused with 50 mL PBS followed by 50 mL 4% (w/v) paraformaldehyde (MilliporeSigma) in PBS. Fixed brains of WT and Trappc9-KO mice were cut into 30 μm thick coronal sections on a cryostat (Leica). Sections were placed in cryoprotectant solutions containing 30% ethylene glycol and 30% glycerol prepared in PBS and stored at –20°C until use.

Immunolabeling was performed with floating brain sections as previously described (19, 73). Sections were removed from cryoprotectant solution and washed in PBS at room temperature. Sections were then permeabilized in PBS containing 5% normal donkey serum, 0.2% Triton X-100, and 1% bovine serum albumin for 1 hour and then stained overnight at 4°C with primary antibodies sheep anti-TH (1:1,000, Novus Biologicals, NB300-109), rabbit anti-Vamp2 (1:1,000, Cell Signaling Technology, 13508), mouse anti-Bassoon (1:400, Enzo Life Sciences, SAP7F407), and mouse anti-NeuN (1:500, MilliporeSigma, MAB377). Afterward, sections were incubated with donkey anti-sheep Alexa Fluor 647 (1:500, Jackson ImmunoResearch, 713-605-147), donkey anti-rabbit Alexa Fluor 488 (1:500, Jackson ImmunoResearch, 711-545-152), and donkey anti-mouse Rhodamine Red-X (1:500, Jackson ImmunoResearch, 715-295-151) for 2 hours at room temperature. Cells in brain sections were identified by staining with Hoechst 34580 (Invitrogen). Brain sections were mounted onto glass slides with ProLong Gold (Invitrogen) for microscopy.

Digital images were acquired through an LSM 880 confocal microscope (Carl Zeiss) with the same settings, including pinhole, laser strength, signal gain, and offset. Super-resolution images were captured using 405, 488, 561, and 633 nm lasers. For quantitative analysis of neurotransmitter release sites, *Z*-stack images across 6 layers with a *Z*-interval of 1.12 μm were captured using a 63× oil immersion objective (Plan-Apochromat, NA1.4) from randomly chosen fields in the NAc. Whole section images were acquired by tile scan using a 10× 0.3 objective and subsequently stitched with ZEN Black software. Composite images were presented as best fit and generated with the ZEISS ZEN Blue software. The number of puncta in reconstructed 3D images was analyzed using the Cell Counter plugin in ImageJ/Fiji software v2.9.0. For analyzing the colocalization of Vamp2, Bassoon, and TH, images from 6 randomly chosen visual fields in the NAc were examined per brain section.

### Statistics.

Prism v.9.0 (GraphPad Software) was used for statistical analyses. Two-tailed unpaired Student’s *t* test was conducted to compare 2 genotype groups or experimental conditions. All data were expressed as means ± SD. *P* < 0.05 was considered statistically significant.

### Study approval.

Experimental procedures involving animals in this study were reviewed and approved by the Institutional Animal Care and Use Committee of the Shanghai Jiao Tong University, Shanghai, China (A2017029), and of the Massachusetts General Hospital, Boston, Massachusetts, USA (2004N000248).

### Data availability.

All data needed to evaluate the conclusions in the paper are present in the paper, the supplement, or the Supporting Data Values file. RNA-Seq data are deposited at NCBI GEO at the accession number GSE269710 (https://www.ncbi.nlm.nih.gov/geo/query/acc.cgi?acc=GSE269710).

## Author contributions

YL, MU, ES, YK, and AB designed, performed, and analyzed experiments. YL, MU, YK, and ZW provided histological evaluations and data analysis. YL, MD, and XL wrote the manuscript. XL conceived, designed, analyzed, and supervised the overall direction of the study.

## Supplementary Material

Supplemental data

Unedited blot and gel images

Supplemental table 1

Supplemental table 2

Supplemental table 3

Supplemental table 4

Supplemental table 5

Supplemental table 6

Supplemental table 7

Supplemental table 8

Supporting data values

## Version 1 06/18/2024

In-Press Preview

## Version 2 08/08/2024

Electronic publication
