# Supplementary material for: Chronic pharmacologic manipulation of dopamine transmission ameliorates metabolic disturbance in Trappc9-linked brain developmental syndrome
Source: JCI Insight. 2024 Jun 18;9(15):e181339. doi: 10.1172/jci.insight.181339 (PMC11383600; doi:10.1172/jci.insight.181339)
Supplement: Supplemental data [file jciinsight-9-181339-s102.pdf]

Supplementary figures and figure legends

Figure S1

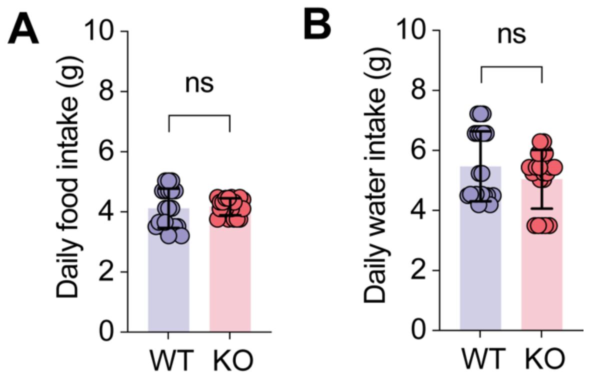

Figure S1 The abrogation of trappc9 in mice does not alter water and food intake. Daily food (**A**) and water (**B**) intake of WT and trappc9 mice were measured for one week at age 5 months (N=12 mice per genotype). Each symbol in bar graphs represents one mouse. Data are Mean±SD. Two-tailed Student's t-test: ns, no significance.

Figure S2

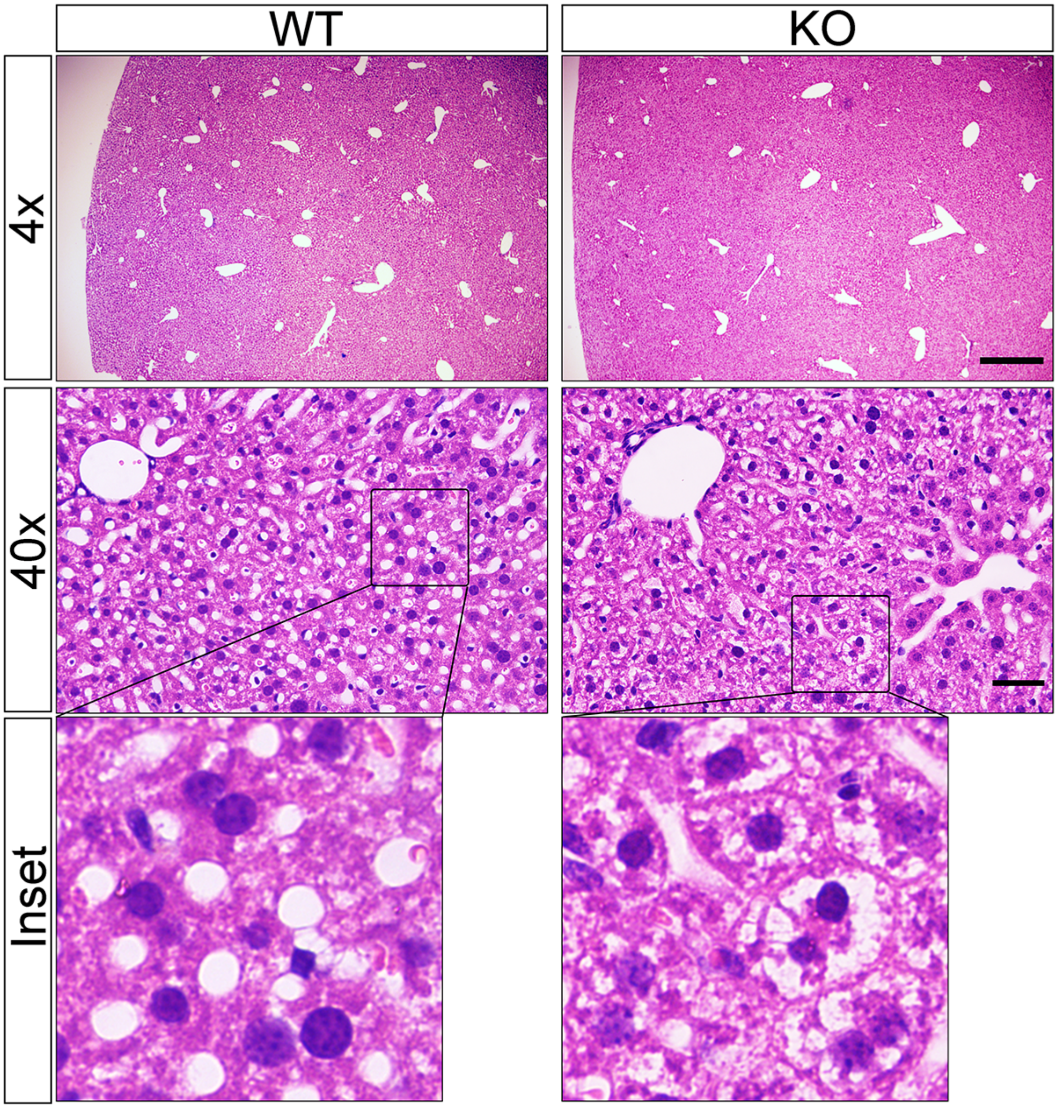

Figure S2 Apparent hepatocyte vacuolation with no infiltration of inflammatory cells in the liver of trappc9 KO mice. Liver sections of trappc9 KO and WT mice were processed for hematoxylin and eosin staining. The age was 3-5 months old. Images were captured with a CCD camera on an Olympus BX53 microscope. Scale bars: 1000 μm and 200 μm for images taken with a 4x objective and a 40x objective, respectively.

Figure S3

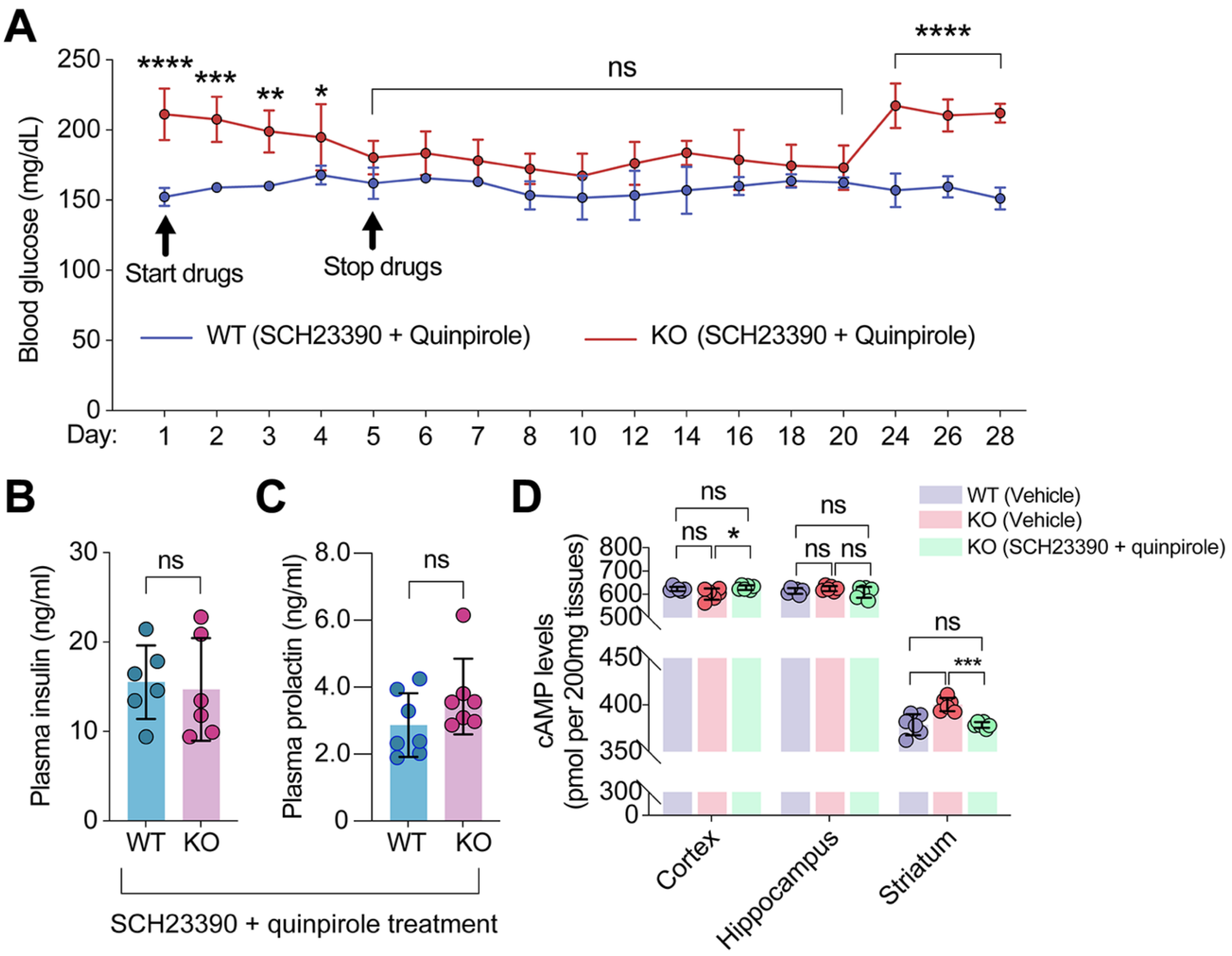

Figure S3 The combined treatment with SCH23390 and quinpirole restores the homeostasis of systemic glucose, insulin and prolactin, and signs of their actions in the brain. A) Efficacy of the SCH23390 and quinpirole combination in lowering blood glucose in trappc9 KO mice. The plot shows that daily administration of both drugs had a trivial effect on blood glucose levels in WT mice, but effectively decreased blood glucose levels in trappc9 KO mice 5 days after treatment. Drug effects lasted for two weeks after the treatment was stopped. Efficacy of the combined treatment in correcting

hyperinsulinemia (**B**) and hyperprolactinemia (**C**) in trappc9 KO mice. **D**) Effects of the treatment combing SCH23390 and quinpirole on cAMP levels in the indicated brain areas. Efficacy studies in (**A**) were done with a cohort of 7 mice for each genotype. Each symbol in bar graphs represents one mouse. Data are Mean±SD. Statistical significance was determined by two-tailed Student's t-test (**A**, **B**, **C**) or one-way ANOVA and post hoc Tukey's test (**D**): * $P<0.05$; ** $P<0.01$; *** $P<0.005$; **** $P<0.001$; ns, no significance.
