# Supplementary material for: Chronic pharmacologic manipulation of dopamine transmission ameliorates metabolic disturbance in Trappc9-linked brain developmental syndrome
Source: JCI Insight. 2024 Jun 18;9(15):e181339. doi: 10.1172/jci.insight.181339 (PMC11383600; doi:10.1172/jci.insight.181339)

Full unedited gel for Figure 5H

Anti-TH

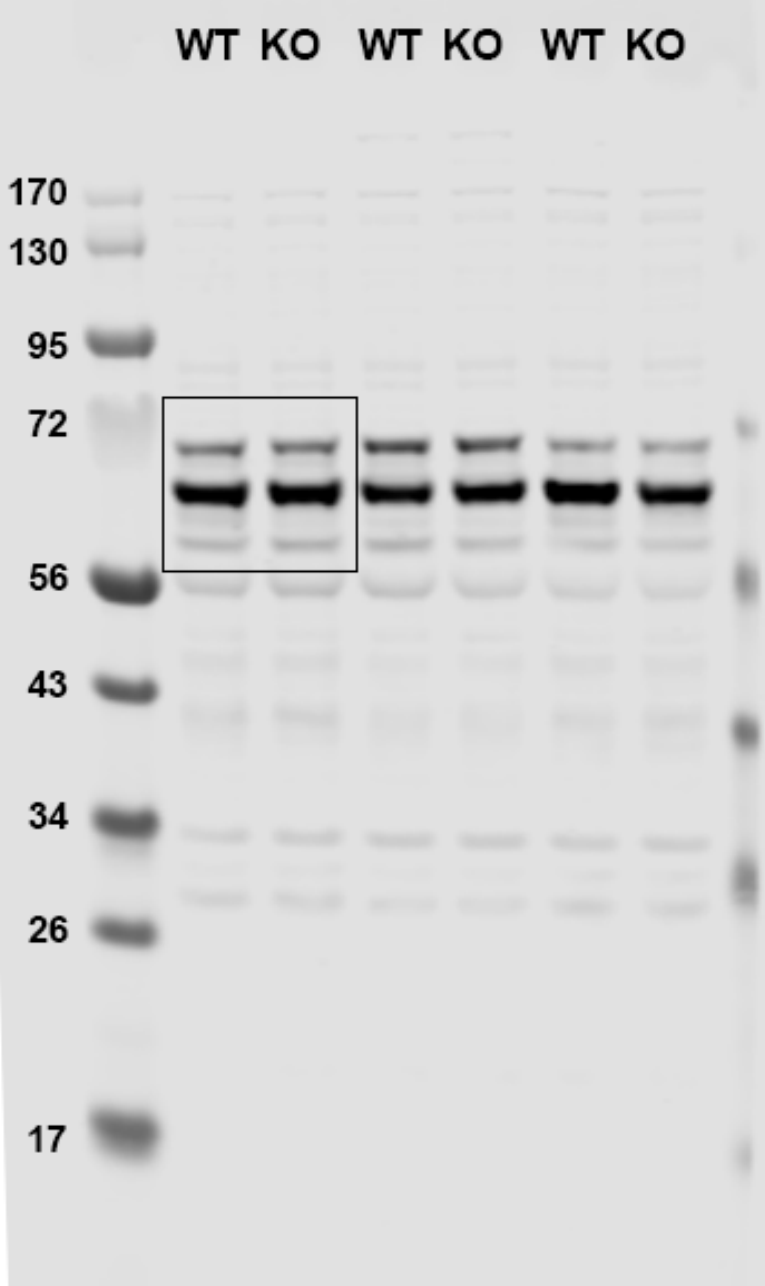

Full unedited gel for Figure 5H

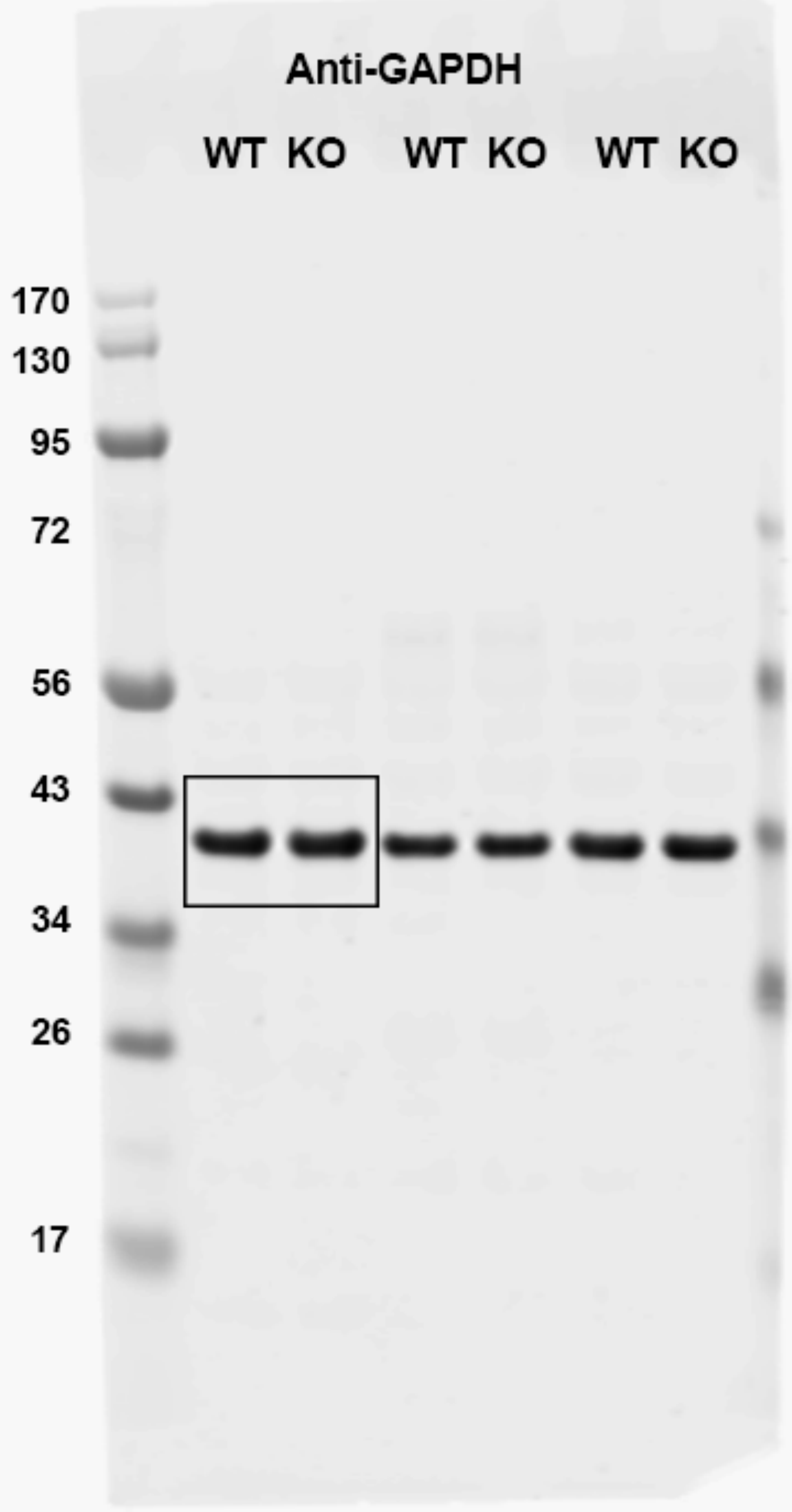

Full unedited gel for Figure6D Anti-Trappc9

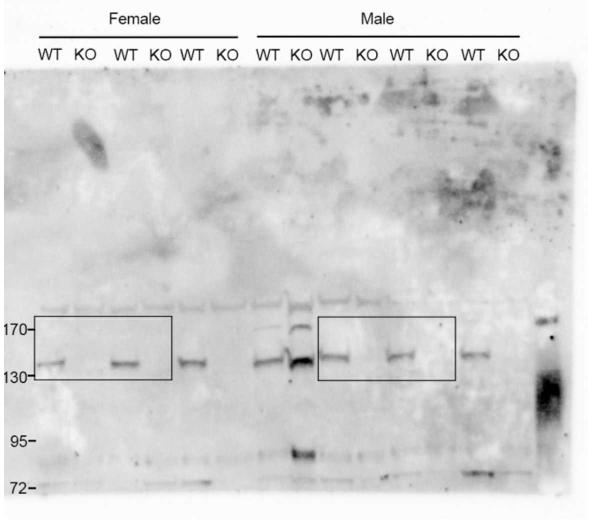

Full unedited gel for Figure6D Anti-Unc13c

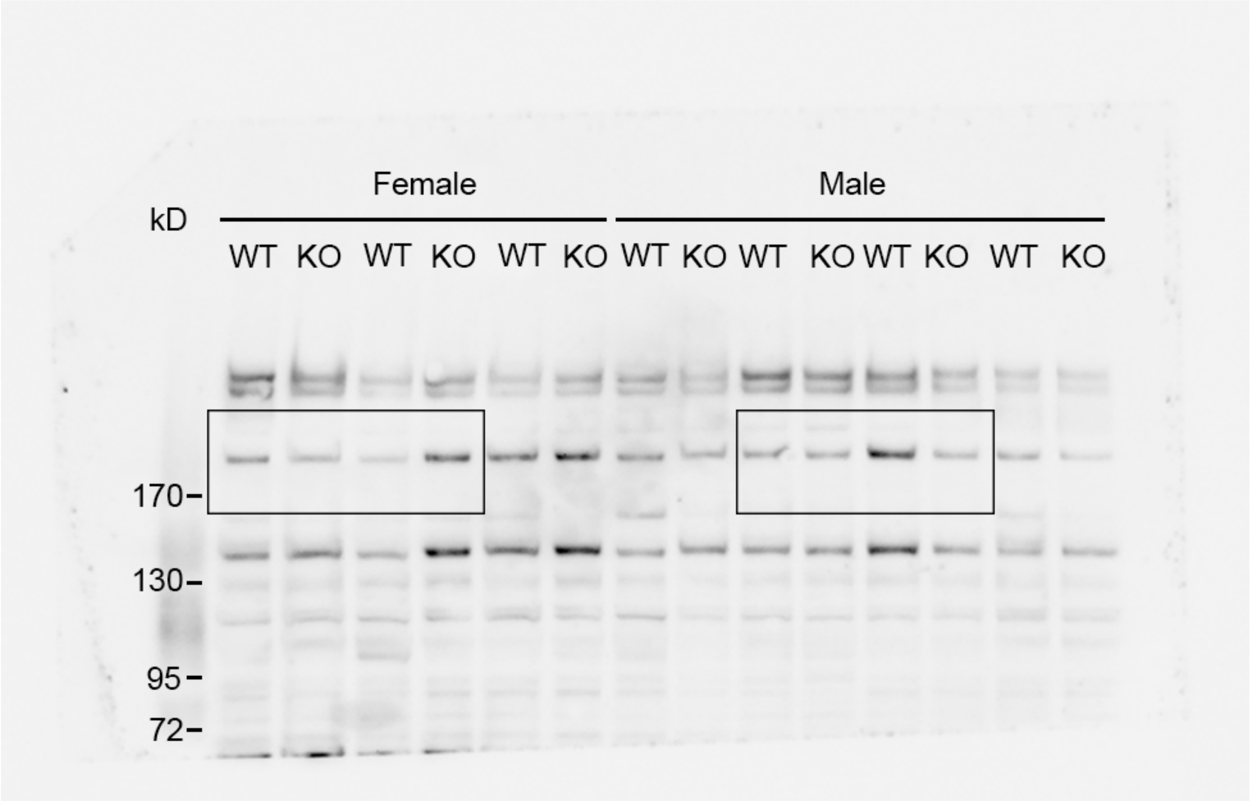

Full unedited gel for Figure6D Anti-Synpo1

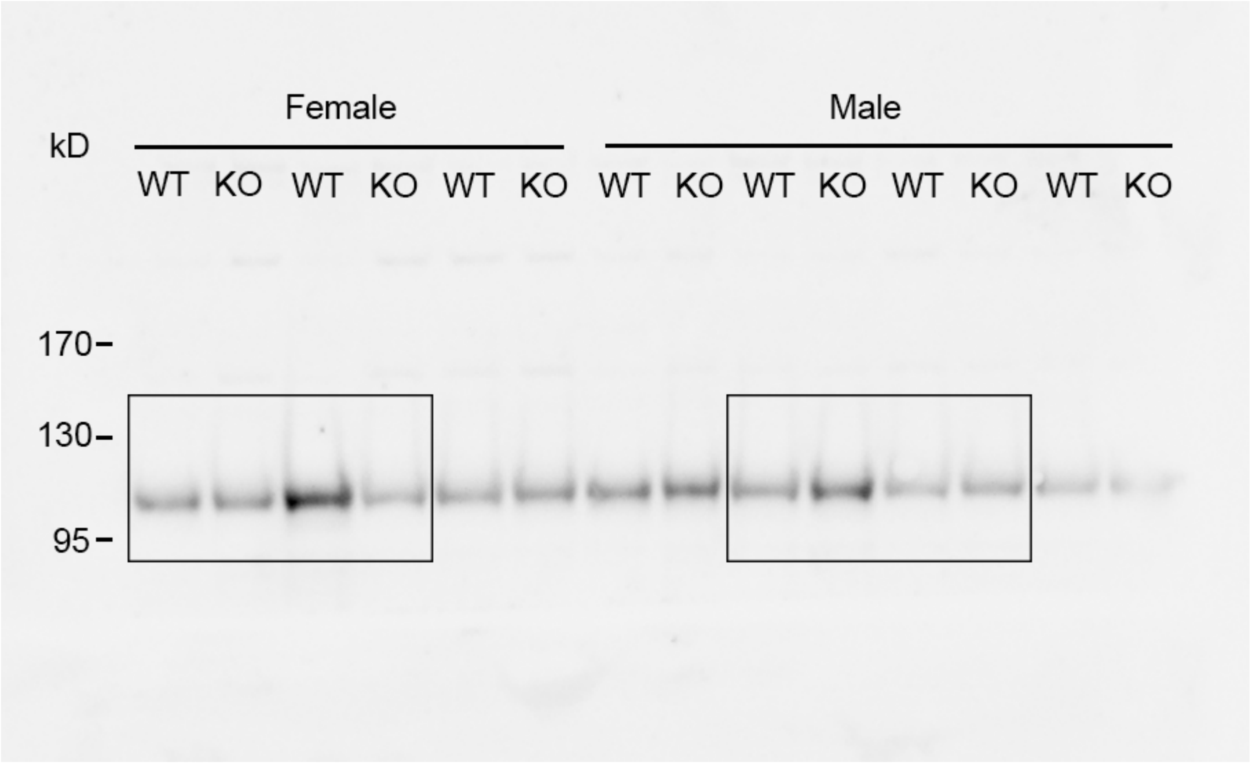

Full unedited gel for Figure6D Anti-Psd95

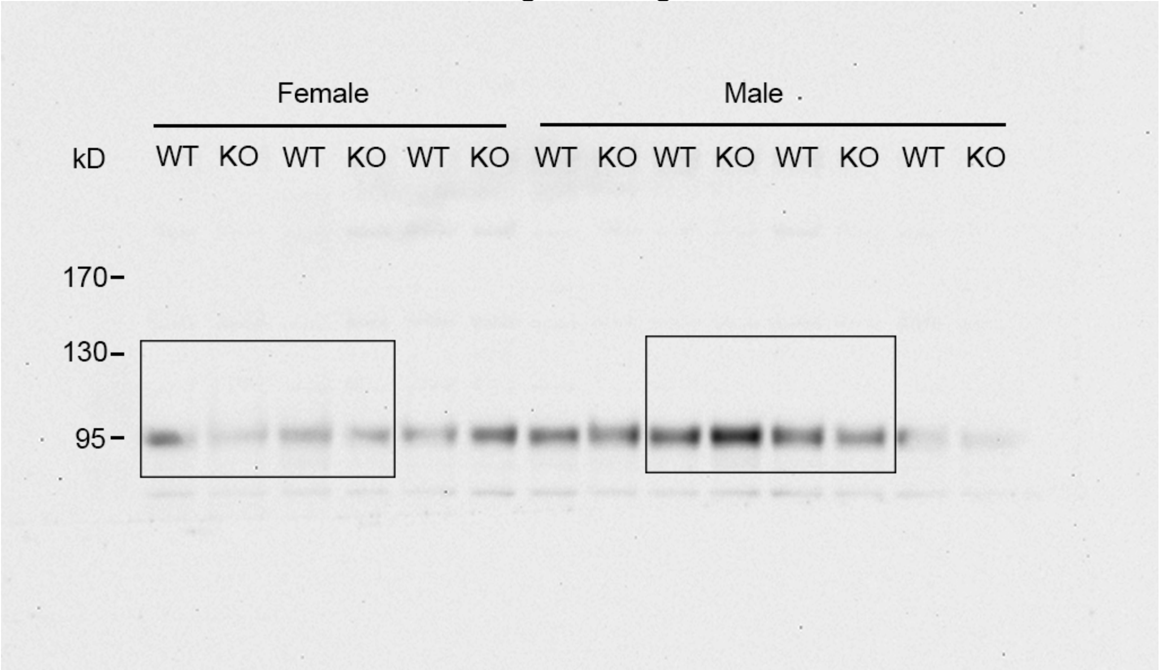

Full unedited gel for Figure6D Anti-Dlg2

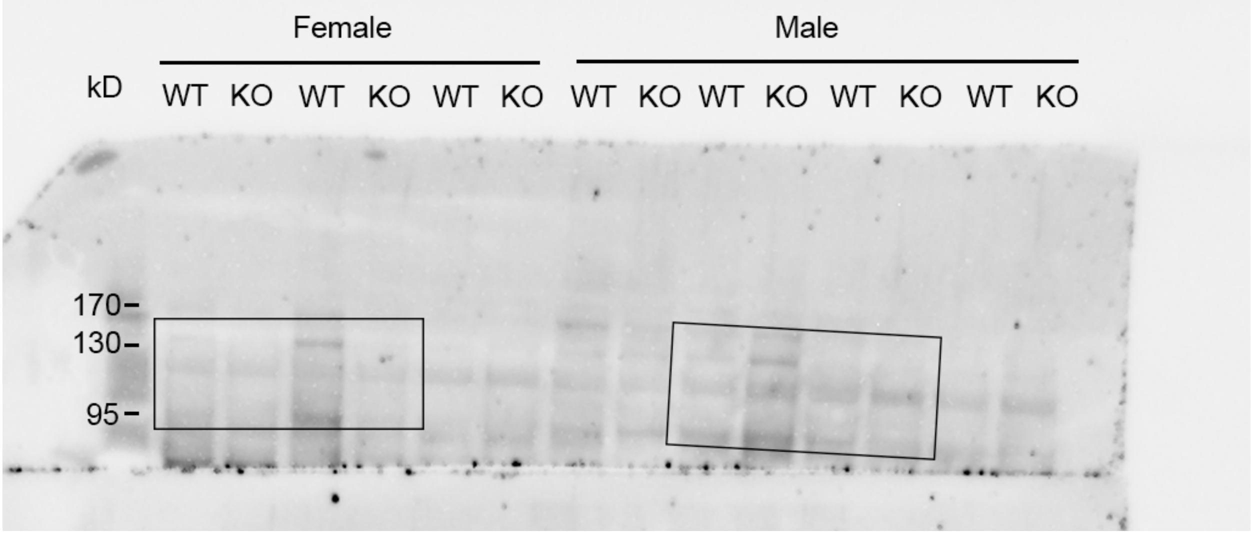

Full unedited gel for Figure6D Anti-Ntsr2

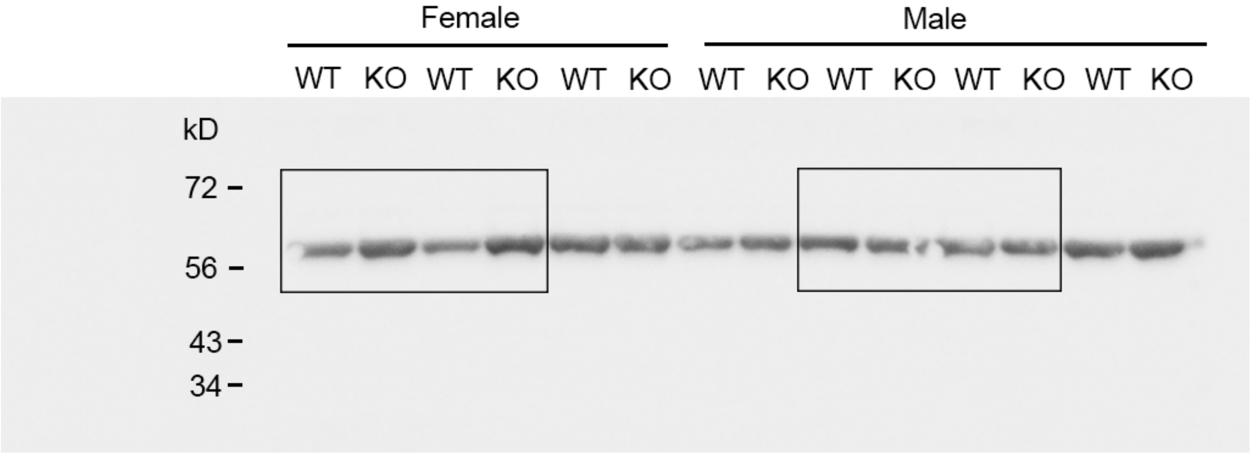

Full unedited gel for Figure6D Anti-Gpm6a

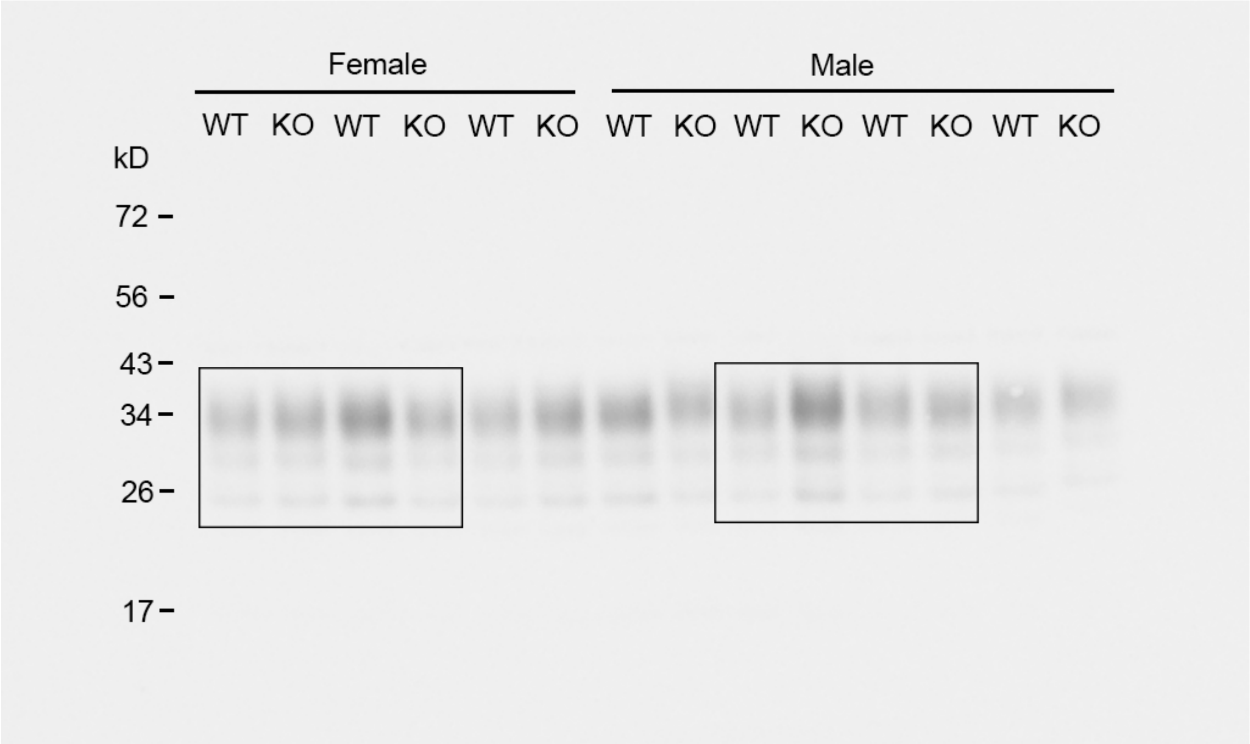

Full unedited gel for Figure6D Anti-Insyn1

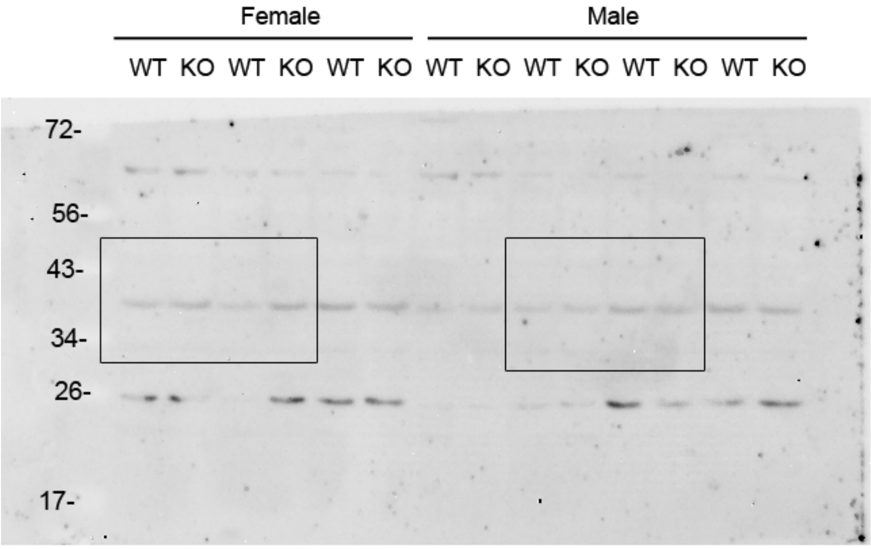

Full unedited gel for Figure6D Anti-GAPDH

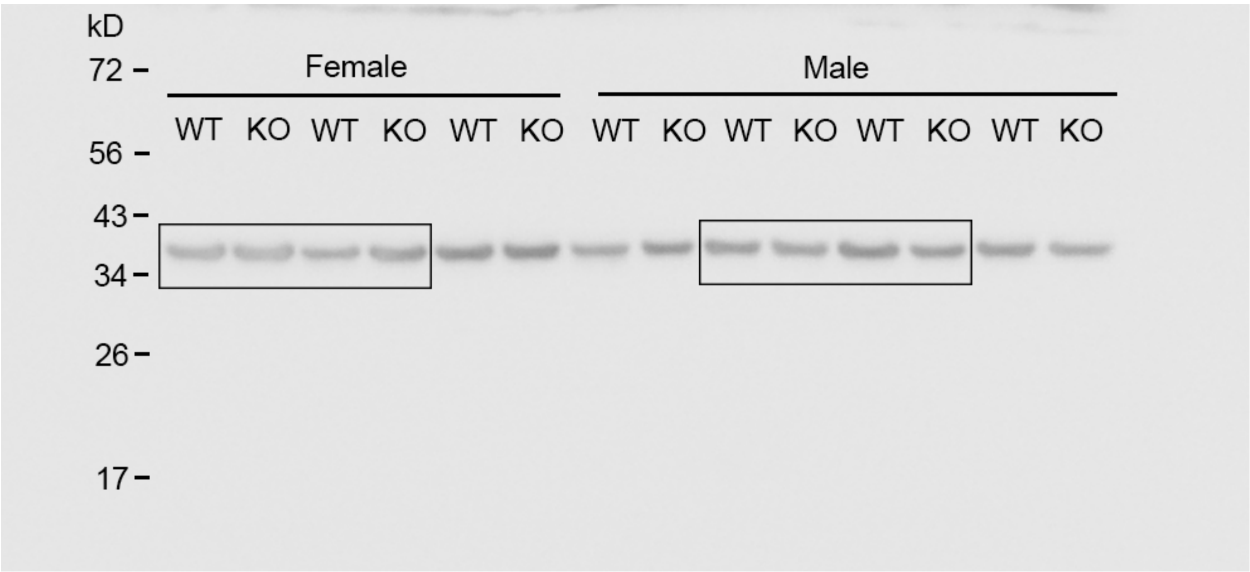

Full unedited gel for Figure6G Anti-Unc13c

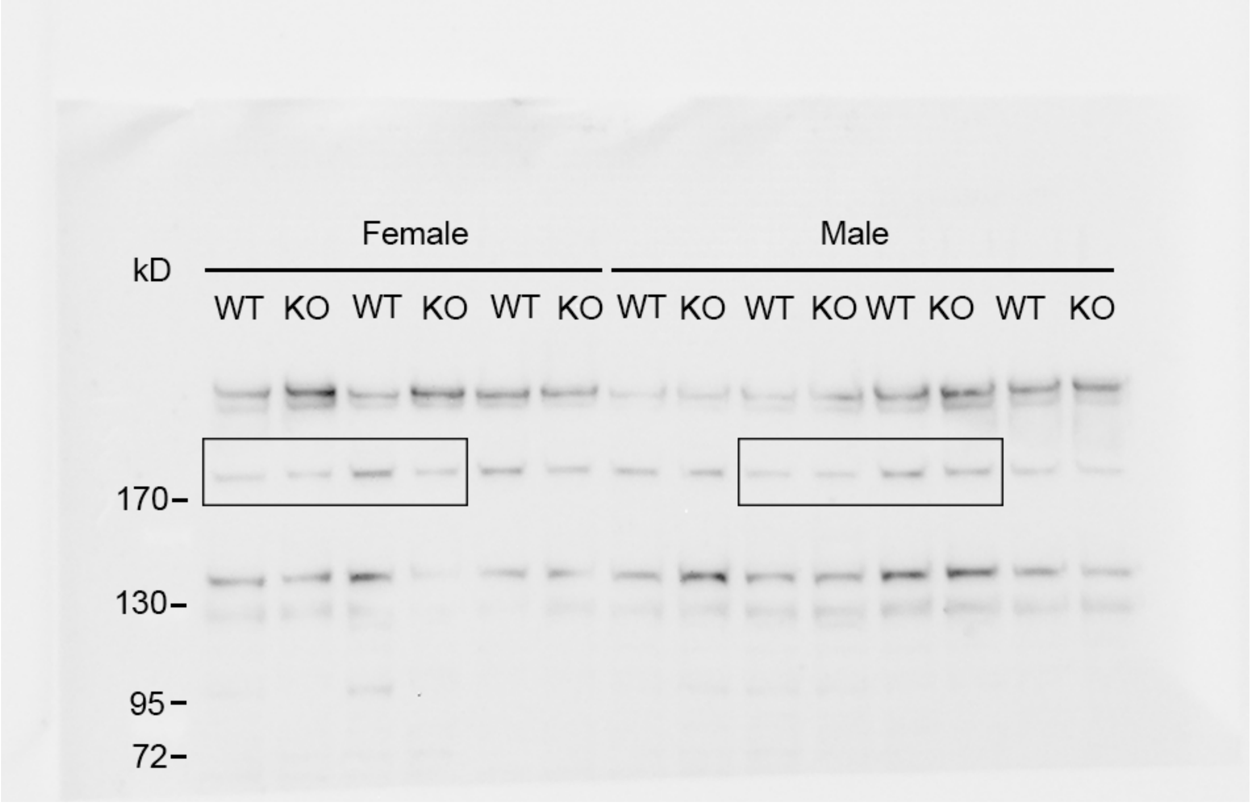

Full unedited gel for Figure6G Anti-Synpo1

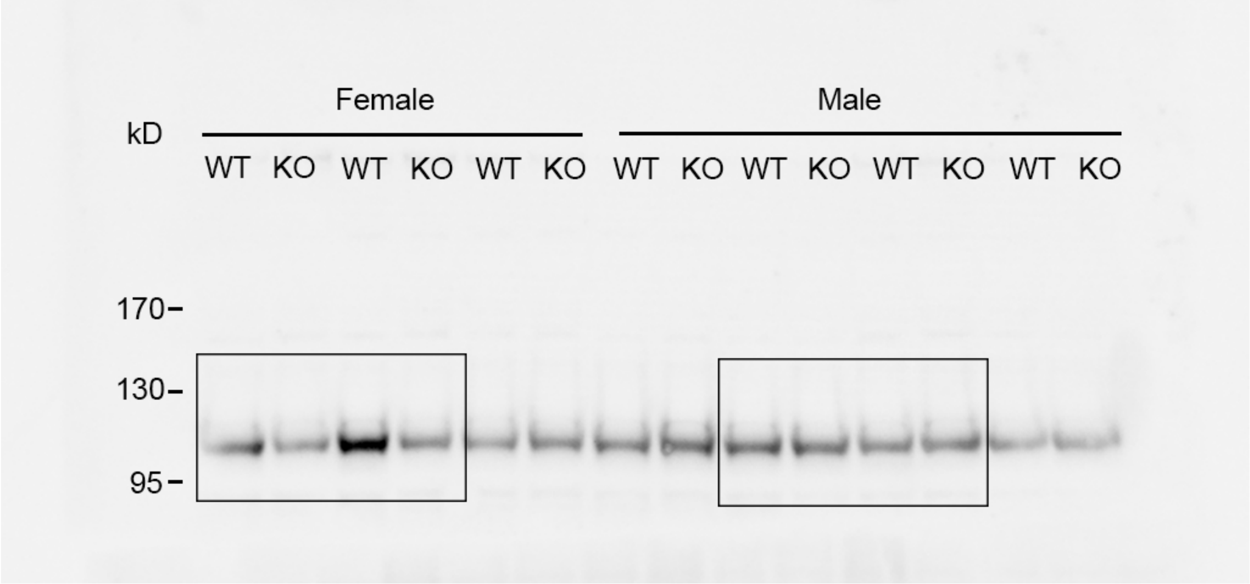

Full unedited gel for Figure6G Anti-Psd95

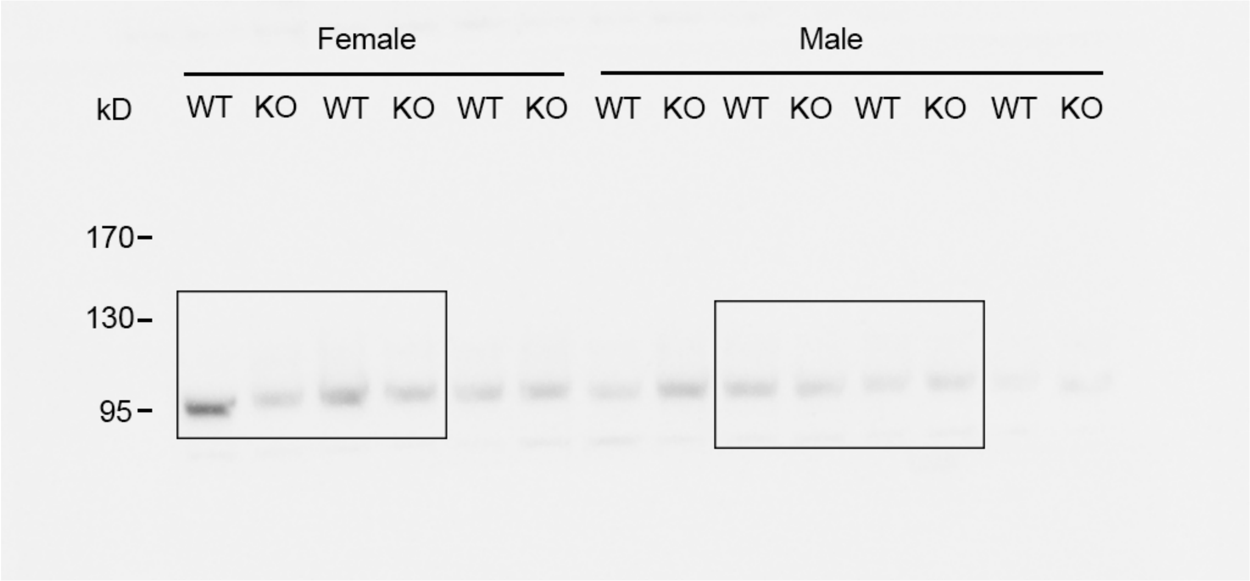

Full unedited gel for Figure6G Anti-Dlg2

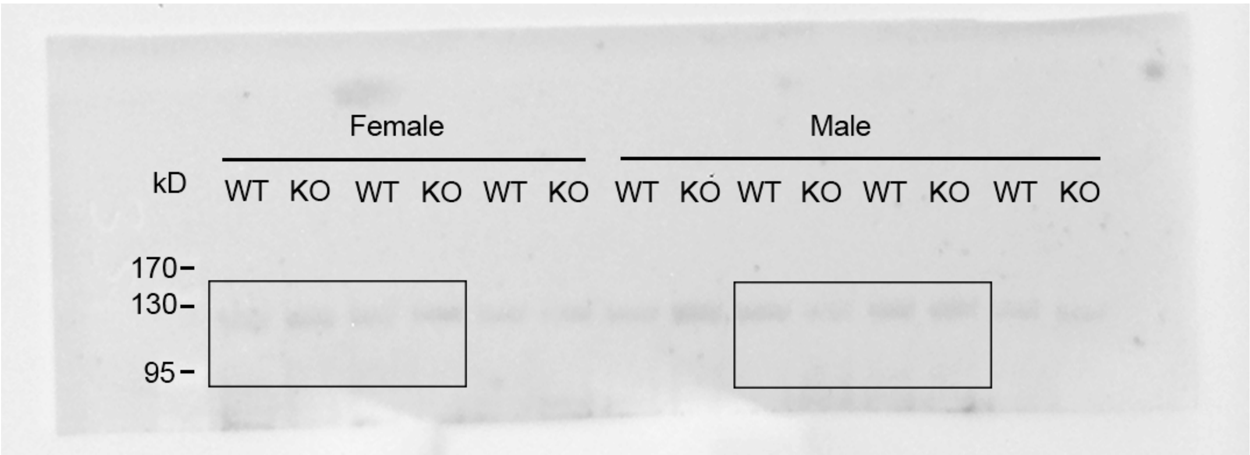

Full unedited gel for Figure6G Anti-Ntsr2

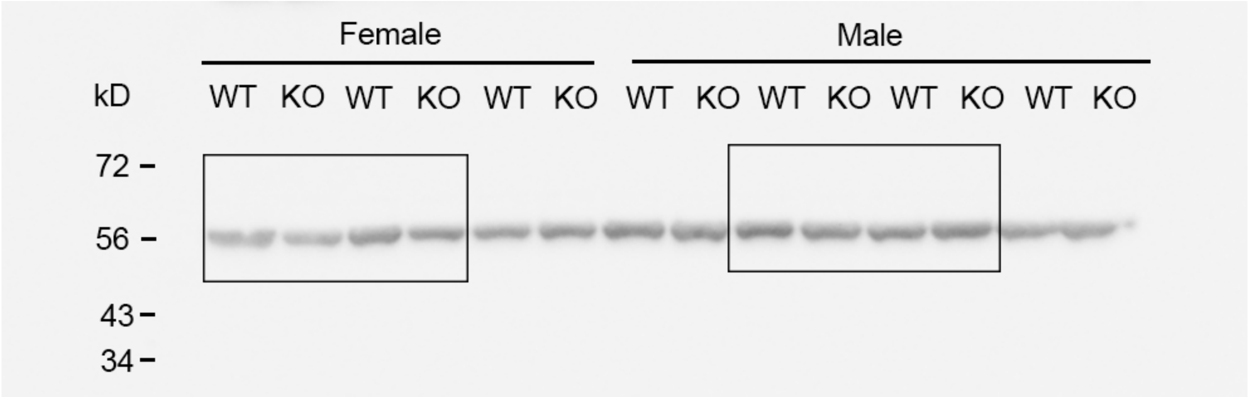

Full unedited gel for Figure6G Anti-Gpm6a

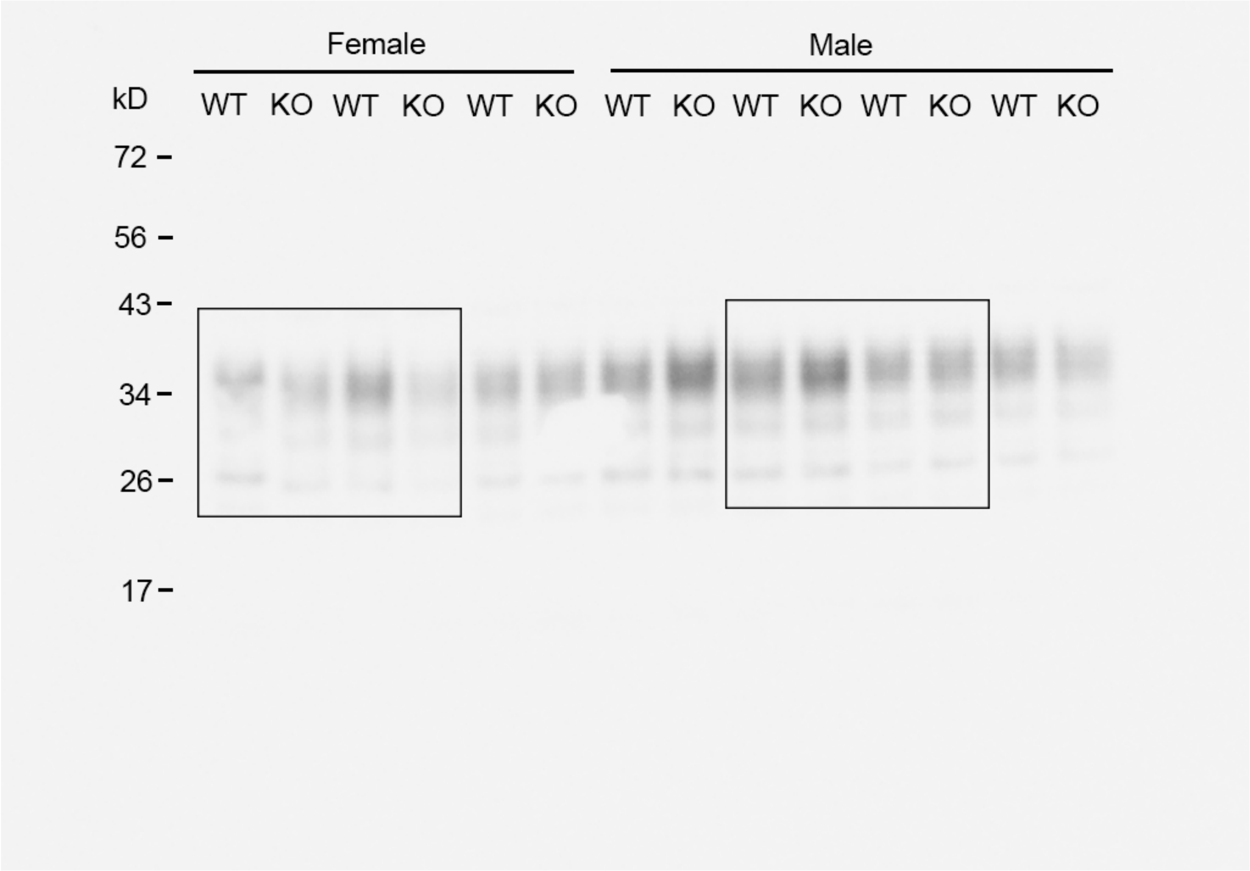

Full unedited gel for Figure6G Anti-Insyn1

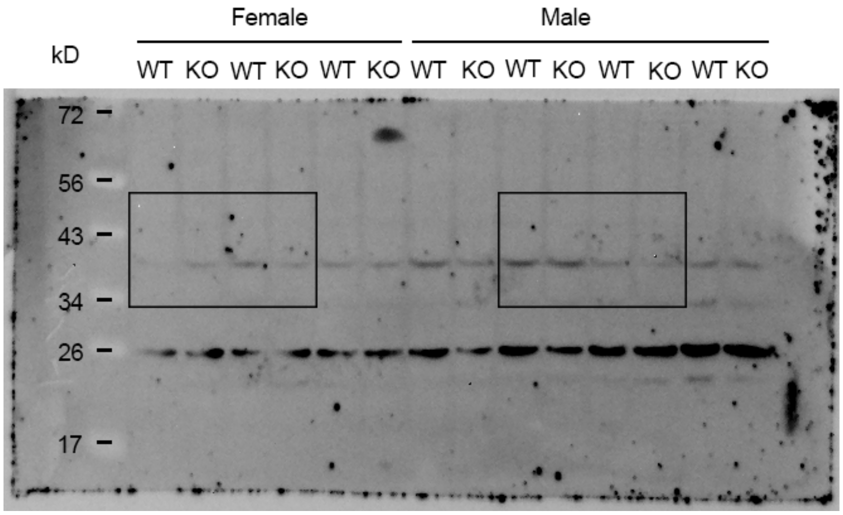

Full unedited gel for Figure6G Anti-GAPDH

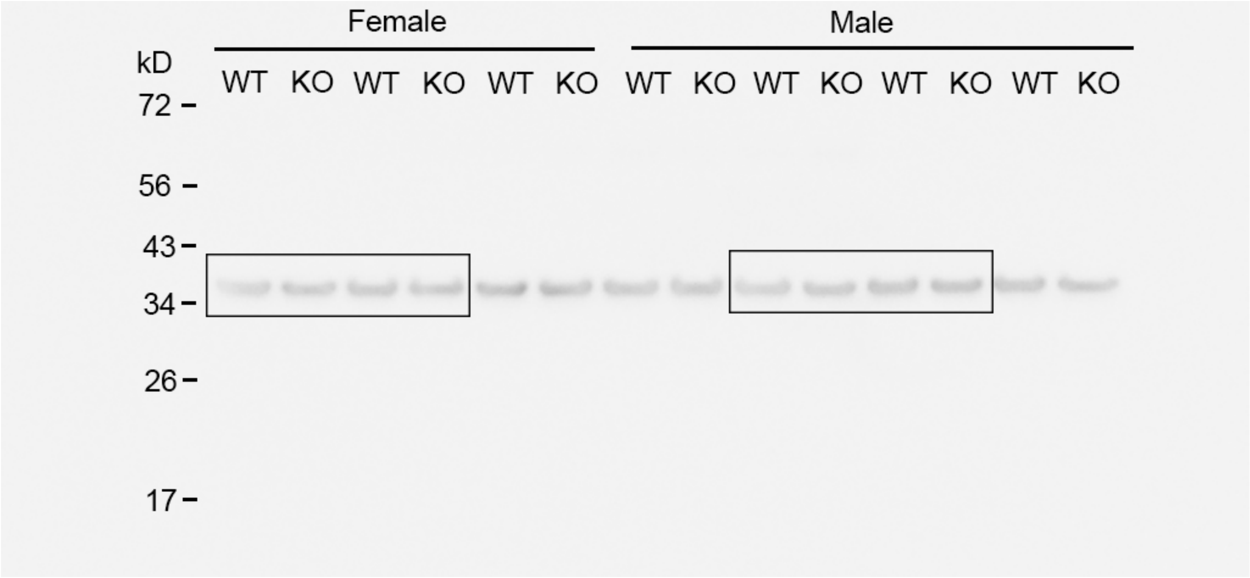

Supplement: Unedited blot and gel images [file jciinsight-9-181339-s103.pdf]
